# Notes on Sarawak (Malaysian Borneo) land snails (Mollusca, Gastropoda, Pulmonata): Thirteen new species, a new genus and a first record of an introduced species

**DOI:** 10.3897/BDJ.14.e188155

**Published:** 2026-05-13

**Authors:** Jaap J. Vermeulen, Camilia Yen Richard, Mohammad Effendi Marzuki, Mohd Zacaery Khalik

**Affiliations:** 1 JK Art and Science, Lauwerbes 8, 2318 AT, Leiden, Netherlands JK Art and Science, Lauwerbes 8, 2318 AT Leiden Netherlands; 2 Faculty of Resource Science and Technology, Universiti Malaysia Sarawak, 94300, Kota Samarahan, Sarawak, Malaysia Faculty of Resource Science and Technology, Universiti Malaysia Sarawak, 94300 Kota Samarahan, Sarawak Malaysia; 3 Institute of Biodiversity and Environmental Conservation, Universiti Malaysia Sarawak, 94300, Kota Samarahan, Sarawak, Malaysia Institute of Biodiversity and Environmental Conservation, Universiti Malaysia Sarawak, 94300 Kota Samarahan, Sarawak Malaysia

**Keywords:** Mollusca, Pulmonata, Malaysia, Sarawak, Borneo

## Abstract

**Background:**

The pulmonates represent an important component of land snail biodiversity in a wide range of habitats across Borneo. Despite their presence in many ecosystems, the diversity of these organisms is still being actively documented through ongoing surveys and taxonomic work. Our study presents 13 new species, a new genus and the first records of pulmonates in Sarawak, contributing to a clearer understanding of species composition in the region.

**New information:**

Thirteen new species of land snails are described from Sarawak, Malaysia (Borneo): *Hemiplecta gambut*, *Vitrinula aulacotropis* (Ariophantidae); *Chloritis platycephala* (Camaenidae); *Kaliella micradelpha*, *K. montismulu*, *K. xystrota*, *Rahula pallidula*, *R. scoliostoma* (Chronidae); *Dyakia nitens*, *Everettia cryptopleura*, *E. micropleura*, *E. platyacris* (Dyakiidae); and *Hypselostoma stenopyrgus* (Hypselostomatidae). The new genus *Gleniskos* (Charopidae) is introduced, two species are transferred; *Gleniskos lafargei* and *G. sarawakensis*. Finally, *Mariaella dussumieri* (Ariophantidae) is reported from Sarawak for the first time.

## Introduction

Our project to produce a handbook on Sarawak land snails leads to the discovery of many novelties. Here, thirteen new species of pulmonate land snails are described. The shells of a West Malaysia species of Charopidae and a Sarawak species described as a Punctidae share coarse radial sculpture. We include both in Charopidae and accommodate them in a new genus *Gleniskos* on account of shared morphological characters which distinguish them from other charopid genera in Malaysia. We also record the introduced ariophantid semi-slug *Mariaella dussumieri* L. Pfeiffer, 1855 for the first time from Borneo. It is known from two distant localities in degraded environments, which suggests that they are more adaptable to various microclimates and are possibly more widespread on Borneo.

## Materials and methods

Material derives from the private collection of the first author (‘JV’), the third author (‘ME’) and the Zoological Museum Universiti Malaysia Sarawak (‘MZU.MOL’), under the care of the last author (‘MZK’). Holotypes are stored in MZU.MOL. A further source is the collection of Han Raven (Wassenaar, the Netherlands, ‘HR’). Descriptions follow format and terminology of Vermeulen and Liew (2022). Pencil drawings are made by the first author, with a Wild M8 stereomicroscope with Camera Lucida. He retains copyright of the illustrations. Photograph credits are given in the captions.

## Taxon treatments

### Hemiplecta gambut sp. nov.

317A91BD-F1BA-544B-BB9E-03FF41F88482

6F996315-84F3-4310-B429-3427954C1042

#### Materials

- **Type status:** Holotype. **Occurrence:** catalogNumber: MZU.MOL 25.29; individualCount: 1; occurrenceID: 2.279546° N, 111.863951° E; **Taxon:** scientificName: *Hemiplecta gambut*; kingdom: Animalia; phylum: Mollusca; class: Gastropoda; family: Ariophantidae; genus: Hemiplecta; scientificNameAuthorship: Albers, 1850; **Location:** country: Malaysia; stateProvince: Sarawak; locality: Bukit Lima ca. 2.6 m SE of Sibu
- **Type status:** Paratype. **Occurrence:** catalogNumber: MZU.MOL 25.58; individualCount: 1; occurrenceID: 1.026474° N, 110.878872° E; **Taxon:** scientificName: *Hemiplecta gambut*; kingdom: Animalia; phylum: Mollusca; class: Gastropoda; family: Ariophantidae; genus: Hemiplecta; scientificNameAuthorship: Albers, 1850; **Location:** country: Malaysia; stateProvince: Sarawak; locality: Simunjan Forest Reserve, Samarahan
- **Type status:** Other material. **Occurrence:** catalogNumber: JV 10083/1; individualCount: 1; occurrenceID: 4.547085° N, 115.157904° E; **Taxon:** scientificName: *Hemiplecta gambut*; kingdom: Animalia; phylum: Mollusca; class: Gastropoda; family: Ariophantidae; genus: Hemiplecta; scientificNameAuthorship: Albers, 1850; **Location:** country: Brunei; stateProvince: Temburong District; locality: Kuala Belalong Field Studies Centre

#### Description

**Figure 1.**
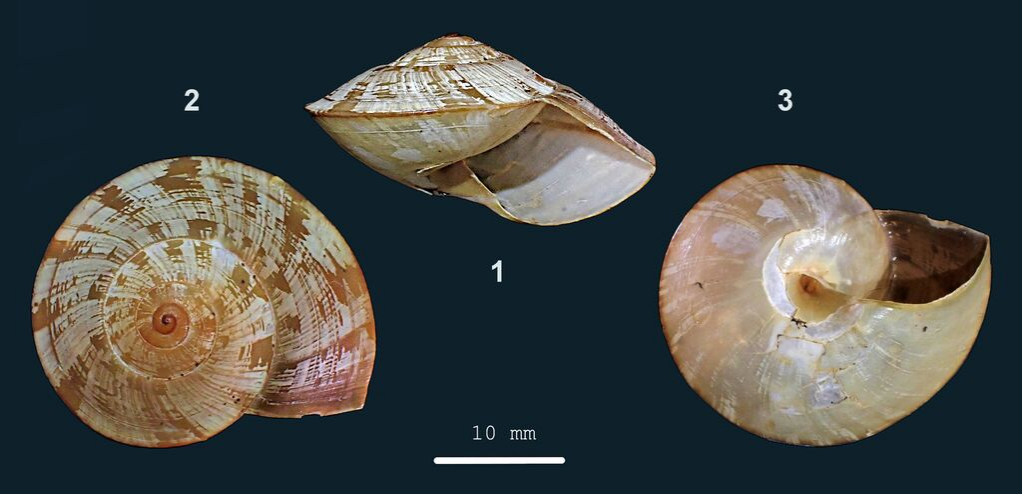
*Hemiplecta gambut* sp. nov.: 1 – holotype (MZU.MOL 25.29), frontal view, shell 19 mm high, 2 –apical view, 3 – umbilical view (photograph MZK).

Shell dextral, large, thin, translucent, (dark) brown-corneous, with oblique, large, opaque, white spots. Surface shiny. Spire depressed-conical, sides approx. flat, apex narrowly rounded. Whorls: Apical whorls convex, other whorls approx. flat, but slightly shouldered towards suture and slightly concave towards periphery; below periphery convex in adults, periphery acutely keeled. Sculpture. Protoconch approx. smooth. Teleoconch. Radial sculpture: Growth lines, raised at uneven intervals. Spiral sculpture: above periphery few widely spaced, wide, shallow, inconspicuous, locally interrupted grooves; below periphery with finer and denser striation. Aperture somewhat quadrangular with lower side narrowly to broadly rounded. Peristome a thin glazing on parietal side, elsewhere not thickened, not spreading. Umbilicus open, narrow, deep. Holotype (Height 19 mm; Width 31 mm). As shown in Fig. 1 (1-3). Dimensions. Height 18.0–19.5 mm; width 30.6–33.7 mm; ratio height/width 0.55–0.59; diameter of first three whorls 1.9–2.6 mm, 4.2–5.9 mm and 8.8–12.3 mm, respectively; umbilicus ca. 1.5 mm wide, ca. 5% of shell width; number of whorls 4 1/2–5 1/8; height aperture 12.0–13.9 mm; width 15.9–18.6 mm.

Animal. Body pale brown, grooves between tubercles darker brown to black. Mantle somewhat lighter brown than foot, spotted ochre-brown; edge with two finger-like lobes.

#### Diagnosis

Resembles *Hemiplecta praeculta* (Smith 1895) in the profile of the last whorl: approx. flat above the periphery, convex below; differs by the pale, opaque blotches on the shell and by the absence of fine oblique sculpture.

#### Etymology

From the Malay ’gambut’ = ‘peat’, referring to the peat forest in which the type was found.

#### Distribution

Sarawak: Samarahan, Sibu. Elevation range: 0–100 m. Primary lowland forest on sandstone/shale bedrock, secondary peat woodland. Also in Brunei. Endemic to Borneo.

### Vitrinula aulacotropis sp. nov.

71BCEC43-CA2F-5AAF-B52F-6224692F66FF

093EFE6A-F752-46D8-90C7-F48647E412BA

#### Materials

- **Type status:** Holotype. **Occurrence:** catalogNumber: MZU.MOL 26.01; occurrenceID: 3.816524° N, 113.771679° E; **Taxon:** scientificName: *Vitrinula aulacotropis*; kingdom: Animalia; phylum: Mollusca; class: Gastropoda; family: Ariophantidae; genus: Vitrinula; scientificNameAuthorship: Gray, 1857; **Location:** country: Malaysia; stateProvince: Sarawak; locality: Niah Caves N.P., Bukit Bekajang
- **Type status:** Paratype. **Occurrence:** catalogNumber: JV 10354/1; occurrenceID: 3.816524° N, 113.771679° E; **Taxon:** scientificName: *Vitrinula aulacotropis*; kingdom: Animalia; phylum: Mollusca; class: Gastropoda; family: Ariophantidae; genus: Vitrinula; scientificNameAuthorship: Gray, 1857; **Location:** country: Malaysia; stateProvince: Sarawak; locality: Niah Cave N.P., S side of limestone area, W of quarry
- **Type status:** Other material. **Occurrence:** catalogNumber: JV 1577/21; occurrenceID: 3.816524° N, 113.771679° E; **Taxon:** scientificName: *Vitrinula aulacotropis*; kingdom: Animalia; phylum: Mollusca; class: Gastropoda; family: Ariophantidae; genus: Vitrinula; scientificNameAuthorship: Gray, 1857; **Location:** country: Malaysia; stateProvince: Sarawak; locality: Niah Caves N.P. N and NW side of limestone massif
- **Type status:** Other material. **Occurrence:** catalogNumber: JV 10399/1; occurrenceID: 3.811388° N, 113.787413° E; **Taxon:** scientificName: *Vitrinula aulacotropis*; kingdom: Animalia; phylum: Mollusca; class: Gastropoda; family: Ariophantidae; genus: Vitrinula; scientificNameAuthorship: Gray, 1857; **Location:** country: Malaysia; stateProvince: Sarawak; locality: Niah Caves N.P. N side of limestone area, Painted Cave
- **Type status:** Other material. **Occurrence:** catalogNumber: JV 10238/14; occurrenceID: 3.817723° N, 113.781411° E; **Taxon:** scientificName: *Vitrinula aulacotropis*; kingdom: Animalia; phylum: Mollusca; class: Gastropoda; family: Ariophantidae; genus: Vitrinula; scientificNameAuthorship: Gray, 1857; **Location:** country: Malaysia; stateProvince: Sarawak; locality: Niah Caves N.P. N side of limestone area, slopes and cliffs along path to Great Cave
- **Type status:** Other material. **Occurrence:** catalogNumber: JV 10296/3; occurrenceID: 3.814792° N, 113.771789° E; **Taxon:** scientificName: *Vitrinula aulacotropis*; kingdom: Animalia; phylum: Mollusca; class: Gastropoda; family: Ariophantidae; genus: Vitrinula; scientificNameAuthorship: Gray, 1857; **Location:** country: Malaysia; stateProvince: Sarawak; locality: Niah Caves N.P. W side of limestone area, path to Bukit Kasut, from base to top
- **Type status:** Other material. **Occurrence:** catalogNumber: MZU.MOL.22.233; otherCatalogNumbers: MZU.MOL.22.250; MZU.MOL.22.256; MZU.MOL.22.258; MZU.MOL.22.298; MZU.MOL.22.299; MZU.MOL.22.561; MZU.MOL.22.563; occurrenceID: 3.822467° N, 113.774051° E; **Taxon:** scientificName: *Vitrinula aulacotropis*; kingdom: Animalia; phylum: Mollusca; class: Gastropoda; family: Ariophantidae; genus: Vitrinula; scientificNameAuthorship: Gray, 1857; **Location:** country: Malaysia; stateProvince: Sarawak; locality: Niah Caves N.P. Great Cave
- **Type status:** Other material. **Occurrence:** catalogNumber: MZU.MOL.25.30; otherCatalogNumbers: MZU.MOL.22.250; MZU.MOL.22.256; MZU.MOL.22.258; MZU.MOL.22.298; MZU.MOL.22.299; MZU.MOL.22.561; MZU.MOL.22.563; occurrenceID: 3.823364° N, 113.762432° E; **Taxon:** scientificName: *Vitrinula aulacotropis*; kingdom: Animalia; phylum: Mollusca; class: Gastropoda; family: Ariophantidae; genus: Vitrinula; scientificNameAuthorship: Gray, 1857; **Location:** country: Malaysia; stateProvince: Sarawak; locality: Niah Caves N.P. Bukit Kasut

#### Description

**Figure 2.**
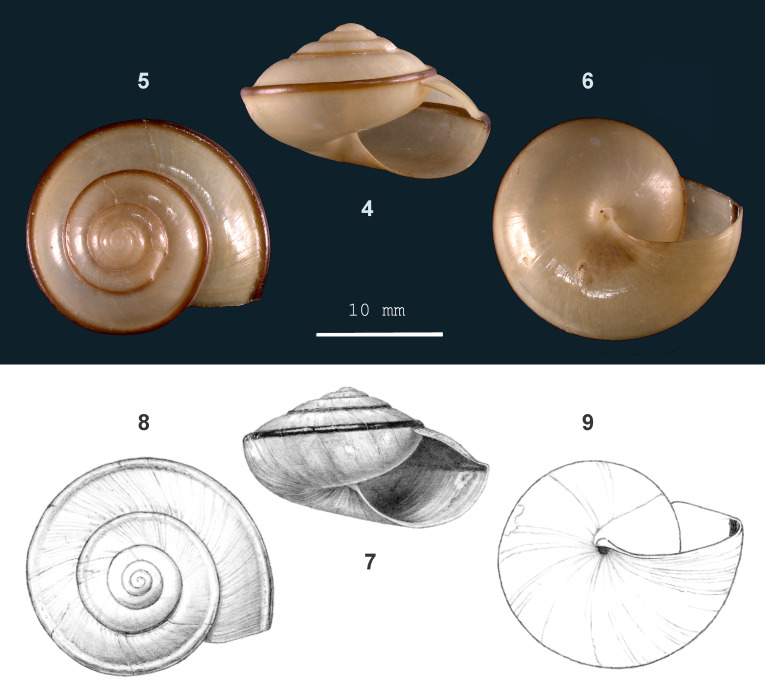
*Vitrinula aulacotropis* sp. nov.: 4 – holotype (MZU.MOL 26.01), frontal view, shell 15 mm high, 5 – apical view, 6 – umbilical view (photograph MZK), 7 – paratype (JV 10354), frontal view, shell 16 mm high, 8 – apical view, 9 – umbilical view.

Shell dextral, large, slightly translucent to opaque, approx. white to greenish-corneous to brown, usually with a narrow (narrower than the unpigmented band above it), pale to dark red-brown band just above periphery; some shells entirely dark red-brown. Surface glossy. Spire depressed-conical, sides flat to slightly convex, apex narrowly rounded. Whorls slightly to moderately convex, last whorl half-way obtusely keeled, with a rather deep furrow above, widely and evenly rounded below. *Sculpture*. Protoconch smooth. Teleoconch. Radial sculpture: Weak growth lines, (locally) slightly raised at uneven intervals. Spiral sculpture mainly on outer whorls, subordinate, inconspicuous (just visible at 40x magnification), localised, very fine striation cutting into crests of raised growth lines. *Aperture* (broadly) crescent-shaped. Peristome a thin glazing on parietal side, elsewhere not thickened, not spreading. *Umbilicus* open, narrow. Holotype (Height 15 mm; Width 24 mm). As shown in Fig. 2 (4-6). Paratype. As shown in Fig. 2 (7-9). Dimensions. Height up to 16.2 mm; width up to 26.7 mm; ratio height/width 0.56–0.63; diameter of first four whorls 1.8–2.3 mm, 3.6–4.2 mm, 6.8–7.9 mm and 12.5–15.3 mm, respectively; umbilicus up to 1.4 mm wide, 3–6% of shell width; number of whorls up to 5 1/8; height aperture up to 11.0 mm; width up to 15.5 mm.

#### Diagnosis

Amongst Bornean *Vitrinula*, it shares an obtusely keeled shell with *V. glutinosa* (Metcalfe 1852) and *V. decrespignyi* (Higgins, 1868), but it differs by the deeper furrow running alongside the keel on the upper surface.

#### Etymology

From αὖλαξ (Ancient Greek) = ‘furrow’ and τρόπις = ‘keel’.

#### Distribution

Sarawak: Niah. Elevation range: 0–400 m. (Disturbed) primary forest on limestone bedrock. Endemic to Sarawak.

### Chloritis platycephala sp. nov.

482DC3C8-0AA5-510E-9903-FB32F7060941

B66F3736-C9B4-4194-AD4B-D6C3D918E67C

#### Materials

- **Type status:** Holotype. **Occurrence:** catalogNumber: MZU.MOL 26.03; occurrenceID: 4.023882° N, 114.822832° E; **Taxon:** scientificName: *Chloritis platycephala*; kingdom: Animalia; phylum: Mollusca; class: Gastropoda; family: Camaenidae; genus: Chloritis ; scientificNameAuthorship: H. Beck, 1837; **Location:** country: Malaysia; stateProvince: Sarawak; locality: Gunung Mulu N.P., Deer Cave, both entrances: plank-walk side and Garden of Eden
- **Type status:** Paratype. **Occurrence:** catalogNumber: JV 10566; occurrenceID: 4.023882° N, 114.822832° E; **Taxon:** scientificName: *Chloritis platycephala*; kingdom: Animalia; phylum: Mollusca; class: Gastropoda; family: Camaenidae; genus: Chloritis ; scientificNameAuthorship: H. Beck, 1837; **Location:** country: Malaysia; stateProvince: Sarawak; locality: Gunung Mulu N.P., Deer Cave, both entrances: plank-walk side and Garden of Eden
- **Type status:** Other material. **Occurrence:** catalogNumber: JV 19379/3; occurrenceID: 4.051556° N, 114.815056° E; **Taxon:** scientificName: *Chloritis platycephala*; kingdom: Animalia; phylum: Mollusca; class: Gastropoda; family: Camaenidae; genus: Chloritis ; scientificNameAuthorship: H. Beck, 1837; **Location:** country: Malaysia; stateProvince: Sarawak; locality: Gunung Mulu N.P., along path from Clearwater Cave to Moonmilk Cave
- **Type status:** Other material. **Occurrence:** catalogNumber: JV 11379/2; occurrenceID: 4.138913° N, 114.893910° E; **Taxon:** scientificName: *Chloritis platycephala*; kingdom: Animalia; phylum: Mollusca; class: Gastropoda; family: Camaenidae; genus: Chloritis ; scientificNameAuthorship: H. Beck, 1837; **Location:** country: Malaysia; stateProvince: Sarawak; locality: Gunung Mulu N.P., Gunung Api, path from camp to pinnacles, incl. pinnacle area

#### Description

**Figure 3.**
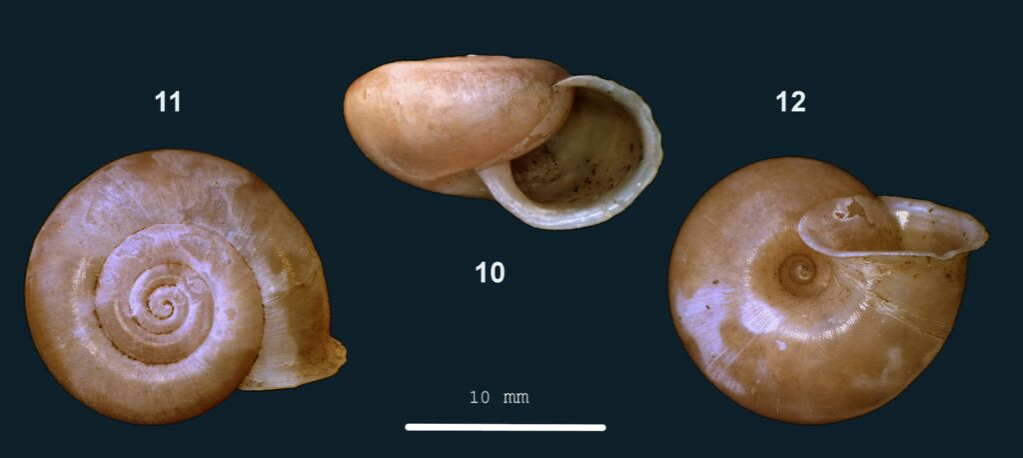
*Chloritis platycephala* sp. nov.: 10 – holotype (MZU.MOL 26.03), frontal view, shell 9.5 mm high, 11 – apical view, 12 – umbilical view (photograph JV).

Shell dextral, medium-sized, thin, opaque, pale ochre-brown, with narrow, darker brown band well above periphery. Surface slightly shiny. Spire almost flat, last half-whorl not or hardly descending; apex flat. Whorls convex, last whorl broadly rounded, more narrowly above periphery and around umbilical impression, slightly shouldered below suture; last whorl with periphery above half its height. Sculpture. Radial sculpture: inconspicuous growth lines, locally raised to dense, low, rounded riblets, particularly below suture. No spiral sculpture. Locally traces of fine, dense granulose-rugulose sculpture visible at 40x magnification. Periostracal hair-scars densely placed on apex and in umbilicus, widely spaced and less conspicuous elsewhere. Aperture obliquely semi-elliptic, almost evenly rounded on palatal and basal side, slightly more narrowly so in basal corner; peristome white, spreading, less so towards upper corner. Umbilicus open, not covered by peristome. Holotype (Height 9.5 mm; Width 17.5 mm). As shown in Fig. 3 (10-12). Dimensions: Height 9.5–10.4 mm; width 17.5–19.4 mm; ratio height/width 0.54–0.56; diameters of first three whorls 2.0–2.4 mm, 4.4–5.0 mm and 9.0–10.0 mm, respectively; last whorl 46–47% of shell width; umbilicus 2.7–3.5 mm wide, 15–18% of shell width; number of whorls 4–4 1/8; height aperture 6.4–6.6 mm; width 7.6–8.5 mm.

#### Diagnosis

Characterised amongst Sarawak *Chloritis* by the almost flat spire, with the last half-whorl not or hardly descending. Resembles *C. plena* (Godwin-Austen, 1891) and *C. meander* (Godwin-Austen, 1891), which also differ by the narrowly rounded palatal side of the aperture.

#### Etymology

From πλατύς (Ancient Greek) = flat and κεφαλή = head, referring to the almost flat spire.

#### Distribution

Sarawak: Mulu. Elevation range: 0–1000 m. (Disturbed) primary forest on limestone bedrock. Endemic to Sarawak.

### Mariaella dussumieri

L. Pfeiffer, 1855

ABCEE830-4DBB-5933-9F76-F00CA324A2E2

Pfeiffer in Gray (1855): 63; Cockerell (1898): 9; Godwin-Austen 1899 (1897–1914): 113, 133; Blanford and Godwin-Austen (1908): 205; Ho (1995): 106; Naggs and Raheem (2000): v, 58; Mordan et al. (2003): (2); Tan and Woo (2010): 66. – Type from India, Mahe (not Mahe, Seychelles, see Cockerell (1898)).
Philippinella moellendorffi auct. Schilthuizen and Liew (2008): 301; Vermeulen and Liew (2022): 214. [Not *Philippinella moellendorffi* Collinge, 1899].

#### Materials

- **Type status:** Other material. **Occurrence:** catalogNumber: HR s.n., d.d. 3/1996; occurrenceID: 4.458358° N, 114.085786° E; **Taxon:** scientificName: *Mariaella dussumieri*; kingdom: Animalia; phylum: Mollusca; class: Gastropoda; family: Ariophantidae; genus: Mariaella; scientificNameAuthorship: Gray, 1855; **Location:** country: Malaysia; stateProvince: Sarawak; locality: Miri

#### Description

**Figure 4.**
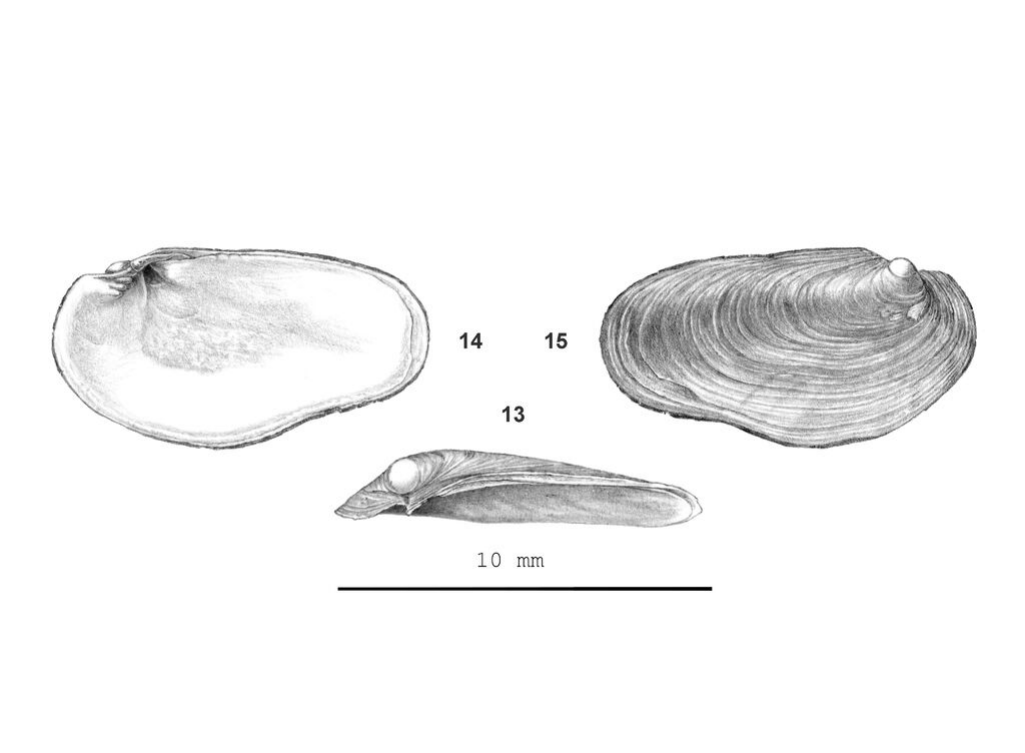
*Mariaella dussumieri*: 13 – (HR s.n., d.d. 3/1996), frontal view, shell 10.1 mm wide, 14 – backside view, 15 – apical view.

Shell thick, white, with thin, green-corneous periostracum, shield-shaped, slightly concave, elliptic to ovate. Apex marginal, close to upper left corner in frontal view, area below apex inside with a sharply outlined, unevenly shaped cavity. Sculpture. Protoconch sharply outlined, smooth. Teleoconch: growth lines only. Representative material. As shown in Fig. 4 (13-15). Dimensions. Short axis up to 5.3 mm; longest up to 10 mm; ratio height/width 0.48–0.53; number of whorls up to 1/4.

#### Distribution

Distribution in Sarawak: Miri. Elevation range: 0–500 m. Degraded environments, fallow land, gardens. Also in Sabah. Distribution elsewhere: India, Sri Lanka, Malaysia (Peninsula), Singapore, Indonesia (Sumatra), Taiwan.

#### Notes

Animals in spirit from Sabah, identified in Schilthuizen and Liew (2008) and Vermeulen and Liew (2022) as *Philippinella moellendorffi* Collinge, 1899, belong here. This introduced species may occur more widespread than is recorded here.

### Gleniskos gen. nov.

05424CDE-E0E5-5C5B-9A03-04D50B8081AD

1893D38D-6E75-45BA-A181-455A914FFDF8

Gleniskos lafargei (Vermeulen & Marzuki, 2014). – ‘Charopa’ lafargei Vermeulen and Marzuki (2014): 31.
Gleniskos sarawakensis (Marzuki, T.-S. Liew & Mohd-Azlan, 2021). – *Paralaoma sarawakensis* Marzuki et al. (2021): 80.
Paralaoma sarawakensis Marzuki, Liew & Mohd-Azlan 2021Marzuki et al. (2021): 80, 36. . Type species.

#### Diagnosis

Amongst West Malaysia genera in Charopidae, it most resembles *Teracharopa* Maassen 2000, in having distinct radial sculpture of well-spaced ribs and very subordinate spiral striation. It differs from *Teracharopa* by the spiral sculpture on protoconch (absent in *Teracharopa*), more widely spaced radial ribs (29–43 on last whorl versus 60–125) and a smaller shell (shell width 1.6–1.7 mm versus 3.7–4.1 mm in shells of 4 1/2–5 whorls).

#### Etymology

Diminutive of γλῆνος (Ancient Greek) = ‘trinklet’.

### Kaliella micradelpha sp. nov.

C2B41F19-E9BD-57B4-9843-A760C9F9C1B1

AEAB973F-12BD-4012-A40A-81373A2D6C3F

#### Materials

- **Type status:** Holotype. **Occurrence:** catalogNumber: MZU.MOL.26.02; occurrenceID: 1.234149° N, 110.416538° E; **Taxon:** scientificName: *Kaliella micradelpha*; kingdom: Animalia; phylum: Mollusca; class: Gastropoda; family: Chronidae; genus: Kaliella; scientificNameAuthorship: Thiele, 1931; **Location:** country: Malaysia; stateProvince: Sarawak; locality: 1^st^ Div., Sungei Bukar headwaters, G. Chupak, East side
- **Type status:** Paratype. **Occurrence:** catalogNumber: JV 14055/146; occurrenceID: 71.234149° N, 110.416538° E; **Taxon:** scientificName: *Kaliella micradelpha*; kingdom: Animalia; phylum: Mollusca; class: Gastropoda; family: Chronidae; genus: Kaliella; scientificNameAuthorship: Thiele, 1931; **Location:** country: Malaysia; stateProvince: Sarawak; locality: 1^st^ Div., Sungei Bukar headwaters, G. Chupak, East side
- **Type status:** Other material. **Occurrence:** catalogNumber: JV 14056/22; occurrenceID: 1.234149° N, 110.416538° E; **Taxon:** scientificName: *Kaliella micradelpha*; kingdom: Animalia; phylum: Mollusca; class: Gastropoda; family: Chronidae; scientificNameAuthorship: Thiele, 1931; **Location:** country: Malaysia; stateProvince: Sarawak; locality: 1^st^ Div., Sungei Bukar headwaters: Gunung Buros S of Gunung Nambi
- **Type status:** Other material. **Occurrence:** catalogNumber: JV 14054/6; occurrenceID: 1.234149° N, 110.416538° E; **Taxon:** scientificName: *Kaliella micradelpha*; kingdom: Animalia; phylum: Mollusca; class: Gastropoda; family: Chronidae; scientificNameAuthorship: Thiele, 1931; **Location:** country: Malaysia; stateProvince: Sarawak; locality: 1st Div., Sungei Bukar headwaters: small limestone hill c. 2 km ESE of Gunung Chupak, W of stream
- **Type status:** Other material. **Occurrence:** catalogNumber: MZU.MOL 24.201/1; occurrenceID: 4.024842° N, 114.824682° E; **Taxon:** scientificName: *Kaliella micradelpha*; kingdom: Animalia; phylum: Mollusca; class: Gastropoda; family: Chronidae; scientificNameAuthorship: Thiele, 1931; **Location:** country: Malaysia; stateProvince: Sarawak; locality: Gunung Mulu N.P.: Lang Cave

#### Description

**Figure 5.**
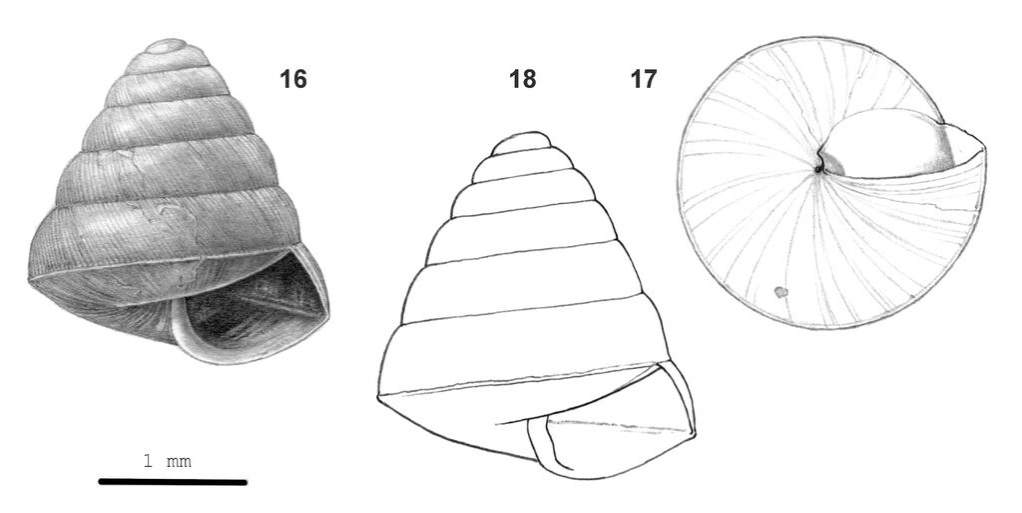
*Kaliella micradelpha* sp. nov.: 16 – holotype (MZU.MOL 26.02), frontal view, shell 2.3 mm high, 17 – same shell, umbilical view, 18 – other paratype shell, frontal view, shell height 2.7 mm.

Shell very small, rather thin, approx. opaque, almost white to dark brown-corneous. Surface with a silky lustre. Spire (somewhat elongated) conical with almost straight or slightly convex sides; apex narrowly rounded. Whorls: Top whorls convex, outer whorls (moderately) convex and often somewhat shouldered below the suture, last whorl somewhat angular and sharply keeled at the periphery, slightly to moderately convex below the periphery. Sculpture. Protoconch with rather densely placed, very fine radial riblets and spiral grooves of approx. equal prominence. Teleoconch. Radial sculpture: Above the periphery very fine, densely and evenly placed, very thin, low riblets, sometimes only locally present; below the periphery, these riblets fade out close to the periphery, elsewhere unevenly spaced growth lines only. Spiral sculpture subordinate, above the periphery often with (traces of) very fine (just visible at 40x magnification), inconspicuous, very dense striation; below the periphery usually with numerous fine (slightly coarser than above the periphery), densely placed, continuous, shallow grooves. Aperture obtusely trapezoid to broadly crescent-shaped, peristome not widened, not thickened. Umbilicus closed or rimate. Holotype (Height 2.3 mm; Width 2.1 mm). As shown in Fig. 5 (16-17). Paratype. As shown in Fig. 5 (18). Dimensions. Height up to 2.8 mm; width up to 2.7 mm; ratio height/width of shells with 5 3/8 whorls or more 1.02–1.13; diameters of the first four whorls 0.3–0.5 mm, 0.6–0.9 mm, 0.9–1.25 mm and 1.3–1.7 mm, respectively; number of whorls up to 6 3/8; height aperture up to 1.1 mm; width up to 1.2 mm.

#### Diagnosis

Amongst Sarawak *Kaliella*, resembles *K. barrakporensis* (Reeve, 1852) and *K. montismulu* sp. nov. They share the peripheral keel and near absence of spiral striation above the periphery. *K. micradelpha* differs by slowly expanding whorls (diameter of 4^th^ whorl 1.4–1.7 mm versus 1.85–3.30 mm). Compared to *K. barrakporensis* (Reeve, 1852), it is smaller (shells of approx. 6 1/4 whorls or more up to 2.7 mm high (versus up to 4.6 mm). *K. angulata* (Issel, 1874) has more rapidly expanding whorls (diameter of 4^th^ whorl 1.9–2.7 mm versus 1.4–1.7 mm) and distinct spiral sculpture.

#### Etymology

From μικρός (Ancient Greek) = ‘small’, and ἀδελφή = ‘sister’.

#### Distribution

Sarawak: Kuching, Mulu. Elevation range: 0–400 m. Primary forest, secondary woods, on limestone bedrock. **Endemic to Sarawak**.

### Kaliella montismulu sp. nov.

2436C25D-5F51-5203-A576-A86208FAA040

98A1DCB7-B595-4383-975E-79A12BA47D12

#### Materials

- **Type status:** Holotype. **Occurrence:** catalogNumber: MZU.MOL 26.04; occurrenceID: 4.051556° N, 114.815056° E; **Taxon:** scientificName: *Kaliella montismulu*; kingdom: Animalia; phylum: Mollusca; class: Gastropoda; family: Chronidae; genus: Kaliella; scientificNameAuthorship: Thiele, 1931; **Location:** country: Malaysia; stateProvince: Sarawak; locality: Gunung Mulu N.P., along path from Clearwater Cave to Moonmilk Cave
- **Type status:** Paratype. **Occurrence:** catalogNumber: JV 10425/5; occurrenceID: 4.051556° N, 114.815056° E; **Taxon:** scientificName: *Kaliella montismulu*; kingdom: Animalia; phylum: Mollusca; class: Gastropoda; family: Chronidae; genus: Kaliella; scientificNameAuthorship: Thiele, 1931; **Location:** country: Malaysia; stateProvince: Sarawak; locality: Gunung Mulu N.P., along path from Clearwater Cave to Moonmilk Cave
- **Type status:** Other material. **Occurrence:** catalogNumber: JV 10240/1; occurrenceID: 3.817723 N, 113.781411° E; **Taxon:** scientificName: *Kaliella montismulu*; kingdom: Animalia; phylum: Mollusca; class: Gastropoda; family: Chronidae; genus: Kaliella; scientificNameAuthorship: Thiele, 1931; **Location:** country: Malaysia; stateProvince: Sarawak; locality: Niah Caves N. P., N side of limestone area, slopes and cliffs along path to Great Cave
- **Type status:** Other material. **Occurrence:** catalogNumber: JV 10429/1; occurrenceID: 4.051556° N, 114.815056° E; **Taxon:** scientificName: *Kaliella montismulu*; kingdom: Animalia; phylum: Mollusca; class: Gastropoda; family: Chronidae; genus: Kaliella; scientificNameAuthorship: Thiele, 1931; **Location:** country: Malaysia; stateProvince: Sarawak; locality: Gunung Mulu N.P., along path from Clearwater Cave to Moonmilk Cave
- **Type status:** Other material. **Occurrence:** catalogNumber: JV 10552/1; occurrenceID: 4.023882° N, 114.822832° E; **Taxon:** scientificName: *Kaliella montismulu*; kingdom: Animalia; phylum: Mollusca; class: Gastropoda; family: Chronidae; genus: Kaliella; scientificNameAuthorship: Thiele, 1931; **Location:** country: Malaysia; stateProvince: Sarawak; locality: Gunung Mulu N.P., Deer Cave, both entrances: plank-walk side and Garden of Eden
- **Type status:** Other material. **Occurrence:** catalogNumber: JV 5620/1; occurrenceID: 4.050038° N, 114.823994° E; **Taxon:** scientificName: *Kaliella montismulu*; kingdom: Animalia; phylum: Mollusca; class: Gastropoda; family: Chronidae; genus: Kaliella; scientificNameAuthorship: Thiele, 1931; **Location:** country: Malaysia; stateProvince: Sarawak; locality: Mulu N.P., entrance of Simons Cave
- **Type status:** Other material. **Occurrence:** catalogNumber: JV 10475/3; occurrenceID: 4.072199° N, 114.876493° E; **Taxon:** scientificName: *Kaliella montismulu*; kingdom: Animalia; phylum: Mollusca; class: Gastropoda; family: Chronidae; genus: Kaliella; scientificNameAuthorship: Thiele, 1931; **Location:** country: Malaysia; stateProvince: Sarawak; locality: Gunung Mulu N.P., entrance to Sarawak Chamber
- **Type status:** Other material. **Occurrence:** catalogNumber: JV 11410/3; occurrenceID: 4.138913° N, 114.893910° E; **Taxon:** scientificName: *Kaliella montismulu*; kingdom: Animalia; phylum: Mollusca; class: Gastropoda; family: Chronidae; genus: Kaliella; scientificNameAuthorship: Thiele, 1931; **Location:** country: Malaysia; stateProvince: Sarawak; locality: Gunung Mulu N.P., Gunung Api, path from camp to pinnacles, incl. pinnacle area
- **Type status:** Other material. **Occurrence:** catalogNumber: JV 5665/2; occurrenceID: 4.138913° N, 114.893910° E; **Taxon:** scientificName: *Kaliella montismulu*; kingdom: Animalia; phylum: Mollusca; class: Gastropoda; family: Chronidae; genus: Kaliella; scientificNameAuthorship: Thiele, 1931; **Location:** country: Malaysia; stateProvince: Sarawak; locality: Gunung Mulu N.P., Gunung Api, path from camp to pinnacles, incl. pinnacle area
- **Type status:** Other material. **Occurrence:** catalogNumber: JV 8003/1; occurrenceID: GPS N/A; **Taxon:** scientificName: *Kaliella montismulu*; kingdom: Animalia; phylum: Mollusca; class: Gastropoda; family: Chronidae; genus: Kaliella; scientificNameAuthorship: Thiele, 1931; **Location:** country: Malaysia; stateProvince: Sabah; locality: Interior Province, Pinangah valley, Batu Urun (= Bukit Sinobang)
- **Type status:** Other material. **Occurrence:** catalogNumber: JV 13013/1; occurrenceID: 6.045970° N, 116.703338° E; **Taxon:** scientificName: *Kaliella monstimulu*; kingdom: Animalia; phylum: Mollusca; class: Gastropoda; family: Chronidae; genus: Kaliella; scientificNameAuthorship: Thiele, 1931; **Location:** country: Malaysia; stateProvince: Sabah; locality: West Coast Province, Kinabalu N.P., Poring Hot Springs, along path to waterfall
- **Type status:** Other material. **Occurrence:** catalogNumber: JV 2502/4; occurrenceID: GPS N/A; **Taxon:** scientificName: *Kaliella montismulu*; kingdom: Animalia; phylum: Mollusca; class: Gastropoda; family: Chronidae; genus: Kaliella; scientificNameAuthorship: Thiele, 1931; **Location:** country: Indonesia; stateProvince: Kalimantan Timur; locality: 30 km W of Balikpapan

#### Description

**Figure 6.**
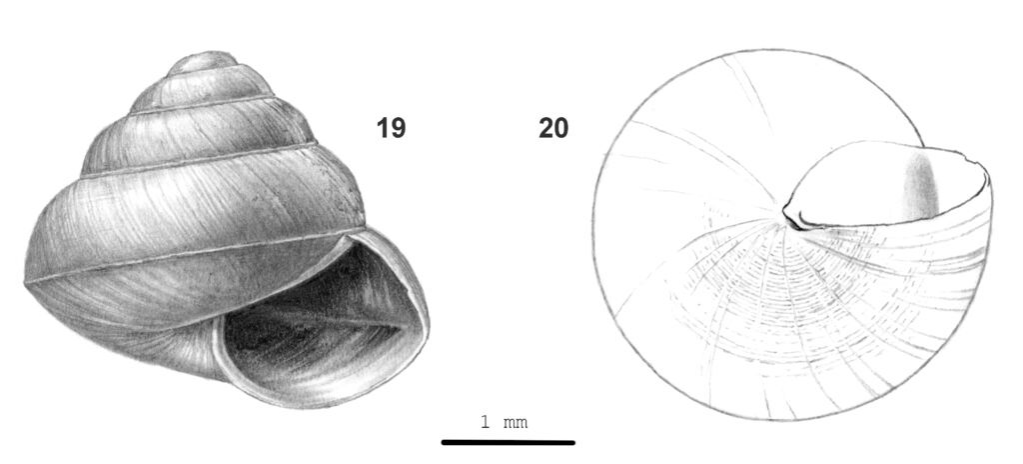
*Kaliella montismulu* sp. nov.: 19 – paratype (JV 10425), frontal view, shell height 3.3 mm, 20 – same shell, umbilical view.

Shell very small, rather thin, somewhat translucent or opaque, (pale) brown-corneous. Surface shiny or with a silky lustre. Spire approx. conical, sides slightly convex; apex narrowly rounded. Whorls convex, last whorl rounded to somewhat angular and sharply keeled at periphery, convex below. Sculpture. Protoconch with rather dense, very fine radial riblets and spiral grooves of approx. equal prominence. Teleoconch. Radial sculpture: Above periphery very fine, dense, evenly placed, very thin, low riblets, often locally present, in some shells absent; below periphery with unevenly spaced growth lines. Spiral sculpture above periphery absent or with (traces of) subordinate, fine (just visible at 40x magnification), inconspicuous, very dense striation; below periphery (locally) with numerous fine, somewhat distant, continuous, shallow grooves. Aperture broadly crescent-shaped, peristome not widened, not thickened. Umbilicus closed, rimate or open, narrow. Holotype (Height 3.3 mm; Width 3.7 mm). As shown in Fig. 6 (19-20). Dimensions. Height up to 3.8 mm; width up to 4.3 mm; ratio height/width of shells with 4 whorls or more 0.8–1.0; diameters of first four whorls 0.60–0.80 mm, 1.2–1.5 mm, 2.00–2.35 mm and 2.9–3.3 mm, respectively; umbilicus up to 0.1 mm wide; number of whorls up to 5 1/2; height aperture up to 1.7 mm; width up to 2.2 mm.

#### Diagnosis

Amongst Sarawak *Kaliella*, resembles *K. barrakporensis* (Reeve, 1852) and *K. micradelpha* sp. nov., all sharing a distinct peripheral keel and near absence of spiral striation above periphery. It differs by its rapidly expanding whorls (diameter of 4^th^ 2.9–3.3 mm versus 1.4–2.7 mm). It also differs from the first by a more inflated appearance, particularly in adults. It differs from *K. accepta* (Smith 1895) (from Sabah and Kalimantan) by its more rapidly expanding whorls (diameter 4^th^ whorl 2.9–3.3 mm versus 1.95–2.5 mm).

#### Etymology

Named after the type locality, with ‘mons’ (Latin) = ‘mountain’.

#### Distribution

Sarawak: Niah, Mulu. Elevation range: 0–300 m. (Disturbed) primary forest on limestone bedrock. Also in Sabah, Kalimantan. Endemic to Borneo.

### Kaliella xystrota sp. nov.

44E72EC9-5749-53D0-A493-8F3BBC089569

39F0648A-B5E9-4225-AF1E-DCBD5454B9B9

#### Materials

- **Type status:** Holotype. **Occurrence:** catalogNumber: MZU.MOL 26.05; occurrenceID: 3.816524° N, 113.771679° E; **Taxon:** scientificName: *Kaliella xystrota*; kingdom: Animalia; phylum: Mollusca; class: Gastropoda; family: Chronidae; genus: Kaliella; scientificNameAuthorship: Thiele, 1931; **Location:** country: Malaysia; stateProvince: Sarawak; locality: Niah Caves N.P., N and NW side of limestone massif
- **Type status:** Paratype. **Occurrence:** catalogNumber: JV 1571/60; occurrenceID: 3.816524° N, 113.771679° E; **Taxon:** scientificName: *Kaliella xystrota*; kingdom: Animalia; phylum: Mollusca; class: Gastropoda; family: Chronidae; genus: Kaliella; scientificNameAuthorship: Thiele, 1931; **Location:** country: Malaysia; stateProvince: Sarawak; locality: Niah Caves N.P., N and NW side of limestone massif
- **Type status:** Other material. **Occurrence:** catalogNumber: JV 10191/2; occurrenceID: 3.817723° N, 113.781411° E; **Taxon:** scientificName: *Kaliella xystrota*; kingdom: Animalia; phylum: Mollusca; class: Gastropoda; family: Chronidae; genus: Kaliella; scientificNameAuthorship: Thiele, 1931; **Location:** country: Malaysia; stateProvince: Sarawak; locality: Niah Caves N.P., N side of limestone area, slopes and cliffs along path to Great Cave
- **Type status:** Other material. **Occurrence:** catalogNumber: JV 20324/1; occurrenceID: 3.816524° N, 113.771679° E; **Taxon:** scientificName: *Kaliella xystrota*; kingdom: Animalia; phylum: Mollusca; class: Gastropoda; family: Chronidae; genus: Kaliella; scientificNameAuthorship: Thiele, 1931; **Location:** country: Malaysia; stateProvince: Sarawak; locality: Niah Caves N.P., S side of limestone area, W of quarry
- **Type status:** Other material. **Occurrence:** catalogNumber: JV 10251/1; occurrenceID: 3.823364° N, 113.762432° E; **Taxon:** scientificName: *Kaliella xystrota*; kingdom: Animalia; phylum: Mollusca; class: Gastropoda; family: Chronidae; genus: Kaliella; scientificNameAuthorship: Thiele, 1931; **Location:** country: Malaysia; stateProvince: Sarawak; locality: Niah Caves N.P., W side of limestone area, path to Bukit Kasut, from base to top

#### Description

**Figure 7.**
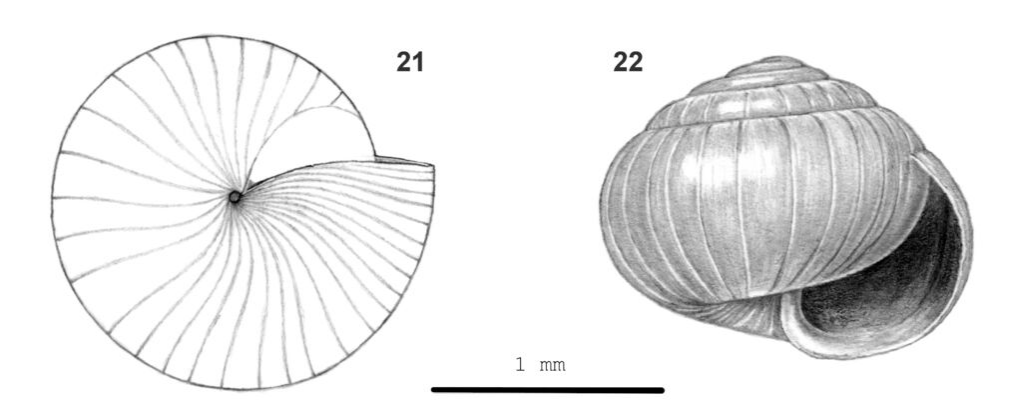
*Kaliella xystrota* sp. nov.: 22 – holotype (MZU.MOL 26.05), frontal view, shell height 1.30 mm, 21 – same shell, umbilical view.

Shell minute, thin, slightly translucent, (pale) corneous to (pale) corneous-brown. Surface glossy. Spire depressed-ovoid, sides moderately convex; apex broadly rounded. Whorls: moderately convex, last whorl broadly rounded, not keeled at periphery, convex below. Sculpture. Protoconch with fine, dense radial riblets and very fine (just visible at 40x magnification), spaced, shallow, spiral grooves. Teleoconch. Radial sculpture orthocline, inconspicuous growth lines and distinct, well-spaced to densely placed, wide, shallow grooves at uneven intervals. Spiral sculpture subordinate, fine, well-spaced, shallow, continuous grooves; sometimes only present on part of shell or absent. Aperture crescent-shaped, peristome not widened nor thickened. Umbilicus approx. closed or open, narrow. Holotype (Height 1.3 mm; Width 1.6 mm). As shown in Fig. 7 (21-22). Dimensions. Height up to 1.5 mm; width up to 1.7 mm; ratio height/width of shells with 3 7/8 whorls or more 0.73–0.88; diameters of first four whorls 0.35–0.40 mm, 0.65–0.80 mm, 0.9–1.2 mm and 1.35–1.60 mm, respectively; umbilicus up to 0.1 mm wide; number of whorls up to 4 7/8; height and width aperture up to 0.9 mm.

#### Diagnosis

Identified amongst Bornean *Kaliella* by the presence of orthocline, distinct, well-spaced to densely placed, wide and shallow grooves at uneven intervals. It may somewhat resemble individuals of *K. oswaldbrakeni* (Marzuki, Liew & Mohd-Azlan, 2021) (as *Microcystina oswaldbrakeni*) (see Marzuki et al. (2021)) with a conspicuous sculpture, but these have a prosocline radial sculpture, which is also more densely placed and finer.

#### Etymology

From Ξυστρωτός (Ancient Greek) = ‘fluted’.

#### Distribution

Sarawak: Niah. Elevation range: 0–400 m. Primary forest, secondary woodland on limestone bedrock. Endemic to Sarawak.

### Rahula pallidula sp. nov.

4F026B1E-1DC8-5D59-AE00-76EF96B163A2

E336290E-DEBE-4C8D-A581-9AA3F8899E26

#### Materials

- **Type status:** Holotype. **Occurrence:** catalogNumber: MZU.MOL 26.06; occurrenceID: GPS N/A; **Taxon:** scientificName: *Rahula pallidula*; kingdom: Animalia; phylum: Mollusca; class: Gastropoda; family: Chronidae; genus: Rahula; scientificNameAuthorship: Godwin-Austen, 1907; **Location:** country: Malaysia; stateProvince: Sarawak; locality: 1^st^ Div., Upper Penrissen valley, G. Kayan, W flank, 1 km N of Kampong Bengoh
- **Type status:** Paratype. **Occurrence:** catalogNumber: JV 13999/37; occurrenceID: GPS N/A; **Taxon:** scientificName: *Rahula pallidula*; kingdom: Animalia; phylum: Mollusca; class: Gastropoda; family: Chronidae; genus: Rahula; scientificNameAuthorship: Godwin-Austen, 1907; **Location:** country: Malaysia; stateProvince: Sarawak; locality: 1^st^ Div., Upper Penrissen valley, G. Kayan, W flank, 1 km N of Kampong Bengoh
- **Type status:** Other material. **Occurrence:** catalogNumber: JV 2540/1; occurrenceID: 1.316726° N, 110.299968° E; **Taxon:** scientificName: *Rahula pallidula*; kingdom: Animalia; phylum: Mollusca; class: Gastropoda; family: Chronidae; genus: Rahula; scientificNameAuthorship: Godwin-Austen, 1907; **Location:** country: Malaysia; stateProvince: Sarawak; locality: Lower Penrissen Valley, Gunung Segu, near Kampong Bunuk
- **Type status:** Other material. **Occurrence:** catalogNumber: JV 13998/1; occurrenceID: GPS N/A; **Taxon:** scientificName: *Rahula pallidula*; kingdom: Animalia; phylum: Mollusca; class: Gastropoda; family: Chronidae; genus: Rahula; scientificNameAuthorship: Godwin-Austen, 1907; **Location:** country: Malaysia; stateProvince: Sarawak; locality: Lower Penrissen Valley, small limestone hill on NW spur of Gunung Rambong

#### Description

**Figure 8.**
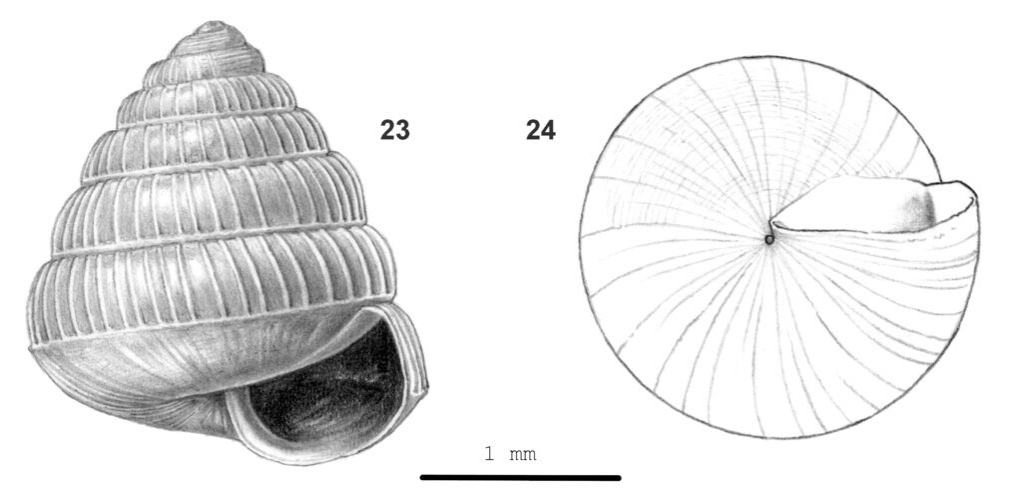
*Rahula pallidula* sp. nov.: 23 – holotype (MZU.MOL 26.06), frontal view, shell height 2.3 mm, 24 – same shell, umbilical view.

Shell very small, rather thick, approx. opaque, green-corneous to white. Surface dull above periphery, shiny below. Spire somewhat elongated conical, sides almost flat, apex somewhat narrowly rounded. Whorls convex, with a thread just above suture, last whorl rounded at periphery, distinctly, narrowly and sharply keeled just below periphery, surface below keel moderately convex. Sculpture. Protoconch with ca. 6 prominent, well-spaced, narrow spiral threads down to the end of second whorl; radial sculpture of very fine, inconspicuous, dense radial riblets. Teleoconch. Radial sculpture: above keel widely, but unevenly spaced (31–49 radial sculptures on last whorl), distinct, slightly prosocline, approx. straight, high, narrow ribs which connect with the keel; interstices smooth or with few growth lines; below keel growth lines only. Spiral sculpture: absent above keel, below keel fine, widely spaced, rather shallow grooves which are partially divided in rows of densely placed pits. Aperture obtusely rectangular, peristome not widened, slightly thickened. Umbilicus open, narrow. Holotype (Height 2.3 mm; Width 2.1 mm). As shown in Fig. 8 (23-24). Dimensions. Height up to 2.6 mm; width up to 2.1 mm; ratio height/width of shells with 5 1/4 whorls or more 1.10–1.30; diameters of first four whorls 0.35–0.50 mm, 0.55–0.90 mm, 0.80–1.25 mm and 1.05–1.45 mm, respectively; umbilicus up to 0.1 mm wide; number of whorls up to 6 1/4; height aperture up to 0.80 mm; width up to 0.95 mm.

#### Diagnosis

Among Bornean *Rahula*, it shares a prominent spiral sculpture on protoconch with *R. raricostulata* (Smith 1893) and *R. scoliostoma* sp. nov. It differs from the first by the pale colour of the shell and by less widely spaced spiral sculpture on the last whorl, below periphery. It differs from the latter species by the less distinctly convex lower surface and by its suprasutural spiral thread along all teleoconch whorls.

#### Etymology

From the diminutive of ‘pallidus’ (Latin) = ‘pale’.

#### Distribution

Sarawak: Serian. Elevation range: 0–300 m. Primary forest, secondary woodland on limestone bedrock. Endemic to Sarawak.

### Rahula scoliostoma sp. nov.

5EE42B6A-8516-55F8-91D4-D63E0688D6CE

183654AE-E96A-4042-95D6-DEDCE5612507

#### Materials

- **Type status:** Holotype. **Occurrence:** catalogNumber: MZU.MOL 26.07; occurrenceID: 1.398319° N, 110.351661° E; **Taxon:** scientificName: *Rahula scoliostoma*; kingdom: Animalia; phylum: Mollusca; class: Gastropoda; family: Chronidae; genus: Rahula; scientificNameAuthorship: Godwin-Austen, 1907; **Location:** country: Malaysia; stateProvince: Sarawak; locality: 1^st^ Div., along road Kuching-Serian, mile 13, the middle one of 3 limestone hills N of the road
- **Type status:** Paratype. **Occurrence:** catalogNumber: JV 11720/4; occurrenceID: 1.398319° N, 110.351661° E; **Taxon:** scientificName: *Rahula scoliostoma*; kingdom: Animalia; phylum: Mollusca; class: Gastropoda; family: Chronidae; genus: Rahula; scientificNameAuthorship: Godwin-Austen, 1907; **Location:** country: Malaysia; stateProvince: Sarawak; locality: 1^st^ Div., along road Kuching-Serian, mile 13, the middle one of 3 limestone hills N of the road

#### Description

**Figure 9.**
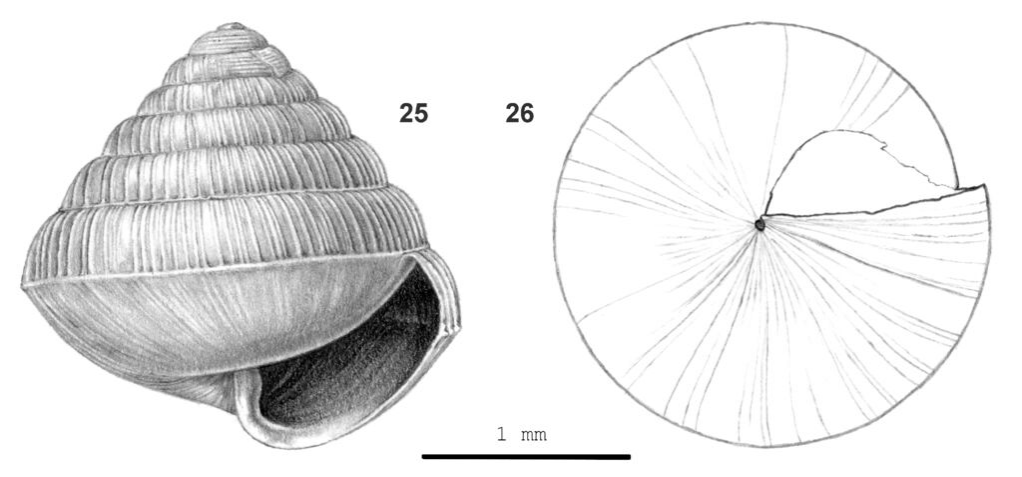
*Rahula scoliostoma* sp. nov.: 25 – holotype (MZU.MOL 26.07), frontal view, shell height 2.5 mm, 26 – same shell, umbilical view.

Shell very small, rather thick, approx. opaque, (pale) brown-corneous. Surface somewhat shiny. Spire conical, sides almost flat, apex somewhat narrowly rounded. Whorls convex, outer with a thread just above suture, last whorl rounded and narrowly and obtusely keeled at periphery, surface below keel distinctly convex. Sculpture. Protoconch with 6–7 prominent, well-spaced, narrow spiral threads down to 1 3/4 whorls; radial sculpture inconspicuous. Teleoconch. Radial sculpture: Above keel rather widely and unevenly spaced (60–73 on last whorl), rather distinct, slightly prosocline, straight, rather low, narrow ribs, which connect with the keel; interstices smooth or with few inconspicuous growth lines; below keel growth lines only. Spiral sculpture: traces of very fine, dense striation above keel, consisting of minute pits, below keel fine, densely placed, rather shallow grooves which are partially divided in rows of densely placed pits. Aperture narrowly and obtusely quadrangular, peristome not widened, not thickened. Umbilicus open, narrow. Holotype (Height 2.5 mm; Width 2.8 mm). As shown in Fig. 9 (25-26). Dimensions. Height up to 2.65 mm; width up to 2.8 mm; ratio height/width of shells with 5 1/2 whorls or more 0.95–0.98; diameters of first four whorls ca. 0.5 mm, 0.75–0.80 mm, 1.10–1.15 mm and 1.55–1.6 mm, respectively; umbilicus up to 0.1 mm wide; number of whorls up to 6 1/2; height aperture up to 1.2 mm; width up to 1.3 mm.

#### Diagnosis

Amongst Bornean *Rahula*, it shares a prominent spiral sculpture on protoconch with *R. raricostulata* (Smith 1893) and *R. pallidula* sp. nov. It differs by having more radial ribs on spire (60–73 on last whorl versus 12–49), by its slightly lower-conical spire (ratio height/width of adult shells 0.95–0.98 (versus 0.98–1.39) and by absence of a suprasutural thread along the inner whorls.

#### Etymology

From σκολιός (Ancient Greek) = ‘bent’ or ‘crooked’ and στόμα = ‘mouth’.

#### Distribution

Sarawak: Serian. Elevation range: 0–100 m. Primary forest, secondary woodland on limestone bedrock. Endemic to Sarawak.

### Dyakia nitens sp. nov.

D0931986-D1C3-51CA-BF5B-61984C511CF5

C162E587-2839-4158-99D4-9996B56879E5

#### Materials

- **Type status:** Holotype. **Occurrence:** catalogNumber: MZU.MOL. 26.08; occurrenceID: 3.811461° N, 113. 787503° E; **Taxon:** scientificName: *Dyakia nitens*; kingdom: Animalia; phylum: Mollusca; class: Gastropoda; family: Dyakiidae; genus: Dyakia; scientificNameAuthorship: Godwin-Austen, 1891; **Location:** country: Malaysia; stateProvince: Sarawak; locality: 4th Div., Niah Caves N.P., Gan Kira
- **Type status:** Paratype. **Occurrence:** catalogNumber: JV 10229/3; occurrenceID: 3.817723° N, 113.781411° E; **Taxon:** scientificName: *Dyakia nitens*; kingdom: Animalia; phylum: Mollusca; class: Gastropoda; family: Dyakiidae; genus: Dyakia; scientificNameAuthorship: Godwin-Austen, 1891; **Location:** country: Malaysia; stateProvince: Sarawak; locality: Niah N.P., N side of limestone area, slopes and cliffs along path to Great Cave
- **Type status:** Other material. **Occurrence:** catalogNumber: JV 1546/1; occurrenceID: 3.816524° N, 113.771679° E; **Taxon:** scientificName: *Dyakia nitens*; kingdom: Animalia; phylum: Mollusca; class: Gastropoda; family: Dyakiidae; genus: Dyakia; scientificNameAuthorship: Godwin-Austen, 1891; **Location:** country: Malaysia; stateProvince: Sarawak; locality: Niah N.P., N and NW side of limestone massif

#### Description

**Figure 10.**
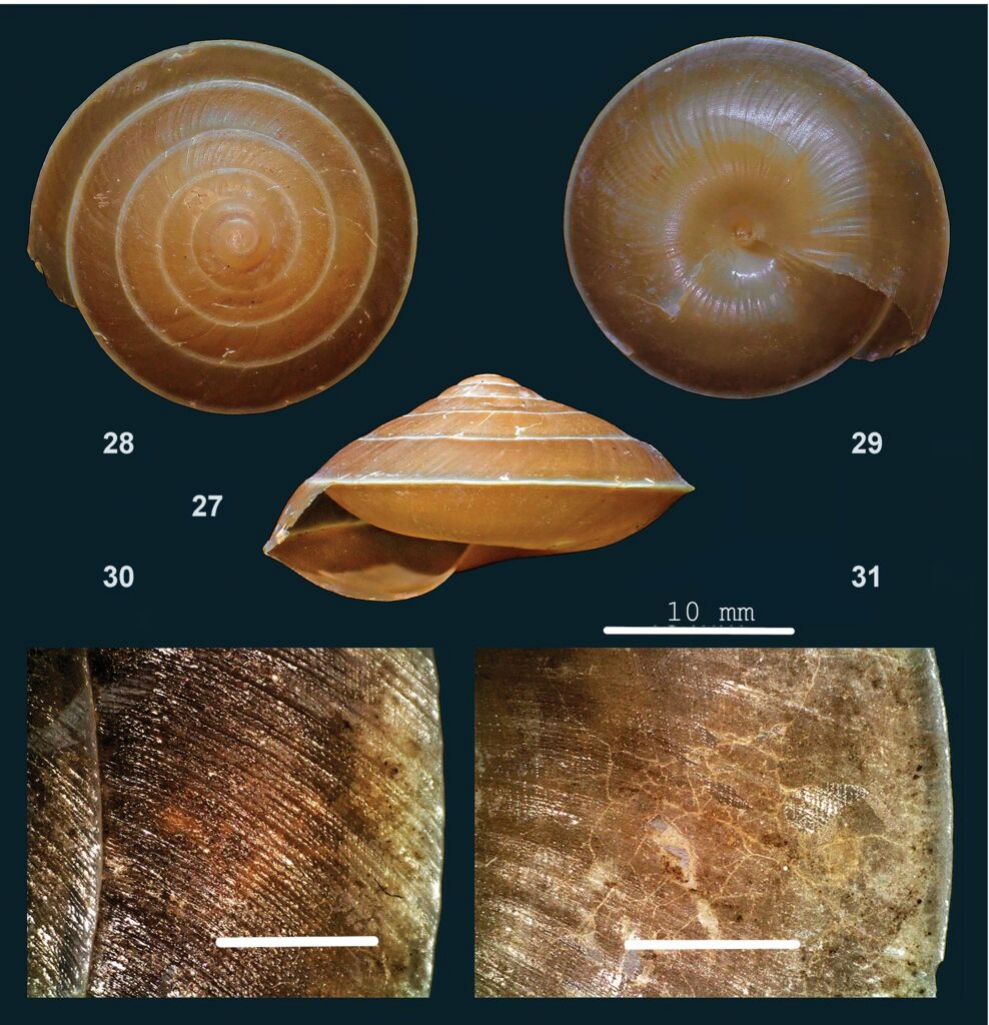
*Dyakia nitens* sp. nov.: 27 – holotype (MZU.MOL 26.08), frontal view, shell height 14 mm, 28 – same shell, apical view, 29 – same shell, umbilical view, 30 – paratype (JV 10229), sculpture above the periphery of last whorl, 31 – same shell, sculpture below the periphery (photographs: 27–29 – MZK; 30–31 – JV, Scale: 1 mm).

Shell sinistral, large, thin, somewhat translucent, corneous-brown, periphery and umbilical impression pale corneous to white. Surface shiny. Spire depressed-conical, sides approx. flat, apex slightly protruding or not, narrowly rounded, last whorl more steeply inclined towards aperture. Whorls: Top whorls moderately convex, outer whorls almost flat, last whorl slightly shouldered towards aperture, moderately convex below; periphery acutely keeled, shell surface slightly impressed immediately above and below. Sculpture. Protoconch with inconspicuous radial riblets. Teleoconch, above periphery. Radial sculpture: Growth lines, at uneven intervals raised or developed into fine, inconspicuous, low riblets, also somewhat finer, somewhat spaced, thin, wavy riblets. Spiral sculpture on outer whorls: locally with inconspicuous, shallow grooves cutting into crests of radial sculpture, creating an inconspicuous granular surface; below periphery, a similar sculpture from periphery to edge of umbilical impression or beyond, but finer and with granules in well-spaced rows. Aperture. Peristome a thin glazing on parietal side, elsewhere not thickened, not spreading. Umbilicus open, deep. Holotype (Height 14 mm; Width 27 mm). As shown in Fig. 10 (27-29). Paratype. As shown in Fig. 10 (30-31). Dimensions. Height up to 17 mm; width up to 30 mm; ratio height/width 0.50–0.55; diameter of first four whorls 2.2–2.9 mm, 4.4–5.5 mm, 7.5–9.7 mm and 14.0–14.8 mm, respectively; number of whorls up to 6 1/4; umbilicus ca. 1 mm wide; height aperture up to 9 mm; width up to 15 mm.

#### Diagnosis

Amongst Bornean *Dyakia*, it shares with *D. hugonis* (Pfeiffer 1864) and *D. intradentata Godwin-Austen 1891* finely granulate sculpture on lower surface of shell, which stretches from periphery to the edge of umbilical depression or beyond (examined at 20x magnification with tangential light) (versus a finely granulate sculpture towards periphery only). It differs from these species by only inconspicuous granular sculpture above periphery, which gives the shell a shiny rather than a silky surface. Additionally, the outer whorls are almost flat (versus moderately convex) above periphery, with last whorls slightly shouldered towards aperture and with shell surface only slightly impressed close to periphery.

#### Etymology

From ‘nitens’ (Latin) = ‘shiny’.

#### Distribution

Sarawak: Niah. Elevation range: 0–300 m. Primary forest on limestone bedrock. Endemic to Sarawak.

### Everettia cryptopleura sp. nov.

686EE17A-7384-5766-B968-A6A1475B93E5

0AAF56A7-D4A0-4DBA-A947-256EF1E30B05

#### Materials

- **Type status:** Holotype. **Occurrence:** catalogNumber: MZU.MOL 26.09; occurrenceID: 82908786-4F31-5F5E-9A3D-9888DD47CBCB; **Taxon:** scientificName: *Everettia cryptopleura*; kingdom: Animalia; phylum: Mollusca; class: Gastropoda; family: Dyakiidae; genus: Everettia; scientificNameAuthorship: Godwin-Austen, 1891; **Location:** country: Malaysia; stateProvince: Sarawak; locality: Bukit Sarang
- **Type status:** Paratype. **Occurrence:** catalogNumber: JV 12867/##; occurrenceID: 2.655167° N, 113.041167° E; **Taxon:** scientificName: *Everettia cryptopleura*; kingdom: Animalia; phylum: Mollusca; class: Gastropoda; family: Dyakiidae; genus: Everettia; scientificNameAuthorship: Godwin-Austen, 1891; **Location:** country: Malaysia; stateProvince: Sarawak; locality: Bukit Sarang
- **Type status:** Other material. **Occurrence:** catalogNumber: JV 13832/20; occurrenceID: 2.99769°N, 113.03684°E; **Taxon:** scientificName: *Everettia cryptopleura*; kingdom: Animalia; phylum: Mollusca; class: Gastropoda; family: Dyakiidae; genus: Everettia; scientificNameAuthorship: Godwin-Austen, 1891; **Location:** country: Malaysia; stateProvince: Sarawak; locality: Lower Tatau River valley, Gua Sidang Sarang
- **Type status:** Other material. **Occurrence:** catalogNumber: JV 12614/4; occurrenceID: A7AA4E72-E1C0-54AB-A61E-DEA657E36424; **Taxon:** scientificName: *Everettia cryptopleura*; kingdom: Animalia; phylum: Mollusca; class: Gastropoda; family: Dyakiidae; genus: Everettia; scientificNameAuthorship: Godwin-Austen, 1891; **Location:** country: Malaysia; stateProvince: Sarawak; locality: Bukit Sarang
- **Type status:** Other material. **Occurrence:** catalogNumber: JV 12866/47; occurrenceID: 2.654254° N, 113.045759° E; **Taxon:** scientificName: *Everettia cryptopleura*; kingdom: Animalia; phylum: Mollusca; class: Gastropoda; family: Dyakiidae; genus: Everettia; scientificNameAuthorship: Godwin-Austen, 1891; **Location:** country: Malaysia; stateProvince: Sarawak; locality: Bukit Anyi, NW-side
- **Type status:** Other material. **Occurrence:** catalogNumber: JV 12867/24; occurrenceID: 2.654254° N, 113.045759° E; **Taxon:** scientificName: *Everettia cryptopleura*; kingdom: Animalia; phylum: Mollusca; class: Gastropoda; family: Dyakiidae; genus: Everettia; scientificNameAuthorship: Godwin-Austen, 1891; **Location:** country: Malaysia; stateProvince: Sarawak; locality: Bukit Anyi, SE-side
- **Type status:** Other material. **Occurrence:** catalogNumber: JV 12868/1; otherCatalogNumbers: JV 19383/25; occurrenceID: 2.655056° N, 113.041272° E; **Taxon:** scientificName: *Everettia cryptopleura*; kingdom: Animalia; phylum: Mollusca; class: Gastropoda; family: Dyakiidae; genus: Everettia; scientificNameAuthorship: Godwin-Austen, 1891; **Location:** country: Malaysia; stateProvince: Sarawak; locality: Bukit Lebik, ground level
- **Type status:** Other material. **Occurrence:** catalogNumber: JV 12869/4; occurrenceID: 2.655161° N, 113.043765° E; **Taxon:** scientificName: *Everettia cryptopleura*; kingdom: Animalia; phylum: Mollusca; class: Gastropoda; family: Dyakiidae; genus: Everettia; scientificNameAuthorship: Godwin-Austen, 1891; **Location:** country: Malaysia; stateProvince: Sarawak; locality: Bukit Lebik, undated sediment deposits c. 40 m above ground level
- **Type status:** Other material. **Occurrence:** catalogNumber: JV 19386/1; occurrenceID: 4.051556° N, 114.815056° E; **Taxon:** scientificName: *Everettia cryptopleura*; kingdom: Animalia; phylum: Mollusca; class: Gastropoda; family: Dyakiidae; genus: Everettia; scientificNameAuthorship: Godwin-Austen, 1891; **Location:** country: Malaysia; stateProvince: Sarawak; locality: Gunung Mulu N.P., along path from Clearwater Cave to Moonmilk Cave

#### Description

**Figure 11.**
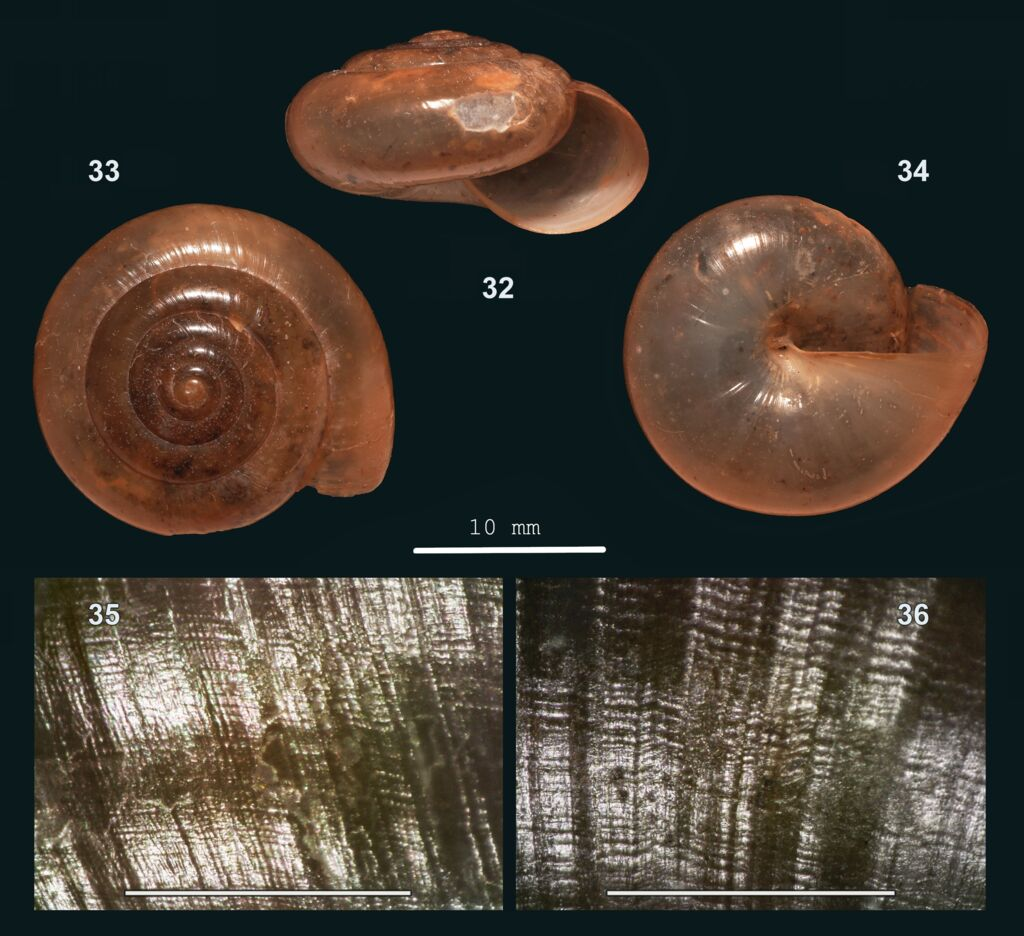
*Everettia cryptopleura* sp. nov.: 32 – holotype (MZU.MOL 26.09), frontal view, shell height 12 mm, 33 – same shell, apical view, 34 – same shell, umbilical view, 35 – paratype (JV 12866), upper surface of last whorl, 36 – same shell, lower surface (photographs: 32-36 - JV, 35-36 Scale: 1 mm).

Shell medium-sized to large, thin, slightly translucent, (pale) corneous, sometimes tinged pale brown. Surface with a silky lustre. Spire slightly raised to depressed-conical, sides flat to slightly convex, apex somewhat narrowly rounded. Whorls slightly to moderately convex; periphery approx. evenly rounded or slightly narrowly rounded. Sculpture. Protoconch locally with inconspicuous, minute, dense radial riblets. Teleoconch. Radial sculpture on last whorl: inconspicuous, somewhat raised growth lines at uneven intervals, which locally are more prominent; lines hardly more prominent towards suture, somewhat curved very close to it or not; next to this (locally), much finer (visible at 40x magnification), dense, interrupted, somewhat braided, low and rounded riblets, particularly above periphery. Spiral sculpture on last whorl: above periphery, localised inconspicuous striation as fine and dense as the finest radial riblets, cutting into their crests, locally dividing them in rows of flattened beads; below periphery, a similar, more prominent striation, approx. continuous and cutting deeper into radial sculpture. Aperture evenly rounded or slightly more narrowly rounded on palatal side. Peristome neither widened, nor thickened. Umbilicus open, narrow, usually not or hardly covered by peristome, sometimes up to half-covered. Holotype (Height 12 mm; Width 20.6 mm). As shown in Fig. 11 (32-34). Paratype. As shown in Fig. 11 (35-36). Dimensions. Height 7.6–14.6 mm; width 13.7–25.0 mm; ratio height/width 0.54–0.64; diameter first four whorls 1.5–2.0 mm, 2.9–3.6 mm, 5.0–6.7 mm and 7.5–10.1 mm, respectively; last whorl 29–39% of shell width; umbilicus 0.4–1.0 mm wide, 3–5% of shell width; number of whorls 5 1/4–6 1/8; height aperture 5.2–10.4 mm, 63–76% of shell height; width 6.6–14.6 mm.

#### Diagnosis

Amongst Sarawak *Everettia*, it shares radial sculpture with *E. micropleura* sp. nov., but differs by localised, densely placed, somewhat braided, flattened radial riblets on outer whorl above periphery (versus *E. micropleura's* slightly more spaced, straight, rounded radial riblets), which are much finer than the growth lines (versus *E. micropleura's* only somewhat finer) and by spiral striation as dense as radial riblets (versus *E. micropleura's* rather widely spaced), locally dividing these into rows of flattened beads (versus *E. micropleura's* elongated sections). It also differs by a wider umbilicus (0.4–1.0 mm diam., 3–5% of shell width (versus *E. micropleura's* up to 0.4 mm, up to 2%). It also shares radial sculpture with *Everettia paulbasintali* Liew, Schilthuizen & Vermeulen, 2009 (from Sabah) (see Liew et al. (2009)), but in the latter, this is coarser and more distinct and it has widely spaced spiral grooves above periphery.

#### Etymology

From κρυπτός (Ancient Greek) = ‘hidden’ and πλευρά = ‘rib’, referring to the inconspicuous microsculpture.

#### Distribution

Sarawak: Mulu (see note), Bukit Sarang, Gua Sidang Sarang (Bintulu). Elevation range: 0–100 m. Primary forest, secondary woodland on limestone bedrock, in more degraded vegetation. Endemic to Sarawak.

#### Notes

1. The fine radial sculpture is best observed with reflecting light parallel to the coiling direction of the whorl and may be only locally present. 2. The Mulu record is based on a single juvenile shell and needs re-assessment.

### Everettia micropleura sp. nov.

70D983C3-064E-5348-92DF-BCB98402593A

47606C9F-6DDE-4975-8E7B-597FB3558250

#### Materials

- **Type status:** Holotype. **Occurrence:** catalogNumber: MZU.MOL 26.10; occurrenceID: 3.823577° N, 113.761899° E; **Taxon:** scientificName: *Everettia micropleura*; kingdom: Animalia; phylum: Mollusca; class: Gastropoda; family: Dyakiidae; genus: Everettia; scientificNameAuthorship: Godwin-Austen, 1891; **Location:** country: Malaysia; stateProvince: Sarawak; locality: Niah N.P.
- **Type status:** Paratype. **Occurrence:** catalogNumber: HR L-1655/1; occurrenceID: 3.823577° N, 113.761899° E; **Taxon:** scientificName: *Everettia micropleura*; kingdom: Animalia; phylum: Mollusca; class: Gastropoda; family: Dyakiidae; genus: Everettia; scientificNameAuthorship: Godwin-Austen, 1891; **Location:** country: Malaysia; stateProvince: Sarawak; locality: Niah N.P.
- **Type status:** Other material. **Occurrence:** catalogNumber: HR s.n., d.d. 9-10-1998/5; occurrenceID: GPS N/A; **Taxon:** scientificName: *Everettia micropleura*; kingdom: Animalia; phylum: Mollusca; class: Gastropoda; family: Dyakiidae; genus: Everettia; scientificNameAuthorship: Godwin-Austen, 1891; **Location:** country: Malaysia; stateProvince: Sarawak; locality: 3 km W of Batu Niah
- **Type status:** Other material. **Occurrence:** catalogNumber: HR L-1596/9; occurrenceID: GPS N/A; **Taxon:** scientificName: *Everettia micropleura*; kingdom: Animalia; phylum: Mollusca; class: Gastropoda; family: Dyakiidae; genus: Everettia; scientificNameAuthorship: Godwin-Austen, 1891; **Location:** country: Malaysia; stateProvince: Sarawak; locality: Batu Niah, 4 km W of locality S93.33
- **Type status:** Other material. **Occurrence:** catalogNumber: HR L-1657/4; occurrenceID: 3.980109° N, 113.948824° E; **Taxon:** scientificName: *Everettia micropleura*; kingdom: Animalia; phylum: Mollusca; class: Gastropoda; family: Dyakiidae; genus: Everettia; scientificNameAuthorship: Godwin-Austen, 1891; **Location:** country: Malaysia; stateProvince: Sarawak; locality: Miri, Sibuti, Mamut, Rumah Baie
- **Type status:** Other material. **Occurrence:** catalogNumber: HR L-1655/2; occurrenceID: 3.980109° N, 113.948824° E; **Taxon:** scientificName: *Everettia micropleura*; kingdom: Animalia; phylum: Mollusca; class: Gastropoda; family: Dyakiidae; genus: Everettia; scientificNameAuthorship: Godwin-Austen, 1891; **Location:** country: Malaysia; stateProvince: Sarawak; locality: Miri, 2 km S of Sibuti
- **Type status:** Other material. **Occurrence:** catalogNumber: HR L-1599/7; occurrenceID: GPS N/A; **Taxon:** scientificName: *Everettia micropleura*; kingdom: Animalia; phylum: Mollusca; class: Gastropoda; family: Dyakiidae; genus: Everettia; scientificNameAuthorship: Godwin-Austen, 1891; **Location:** country: Malaysia; stateProvince: Sarawak; locality: Lambir Hills Palmoil Plantation

#### Description

**Figure 12.**
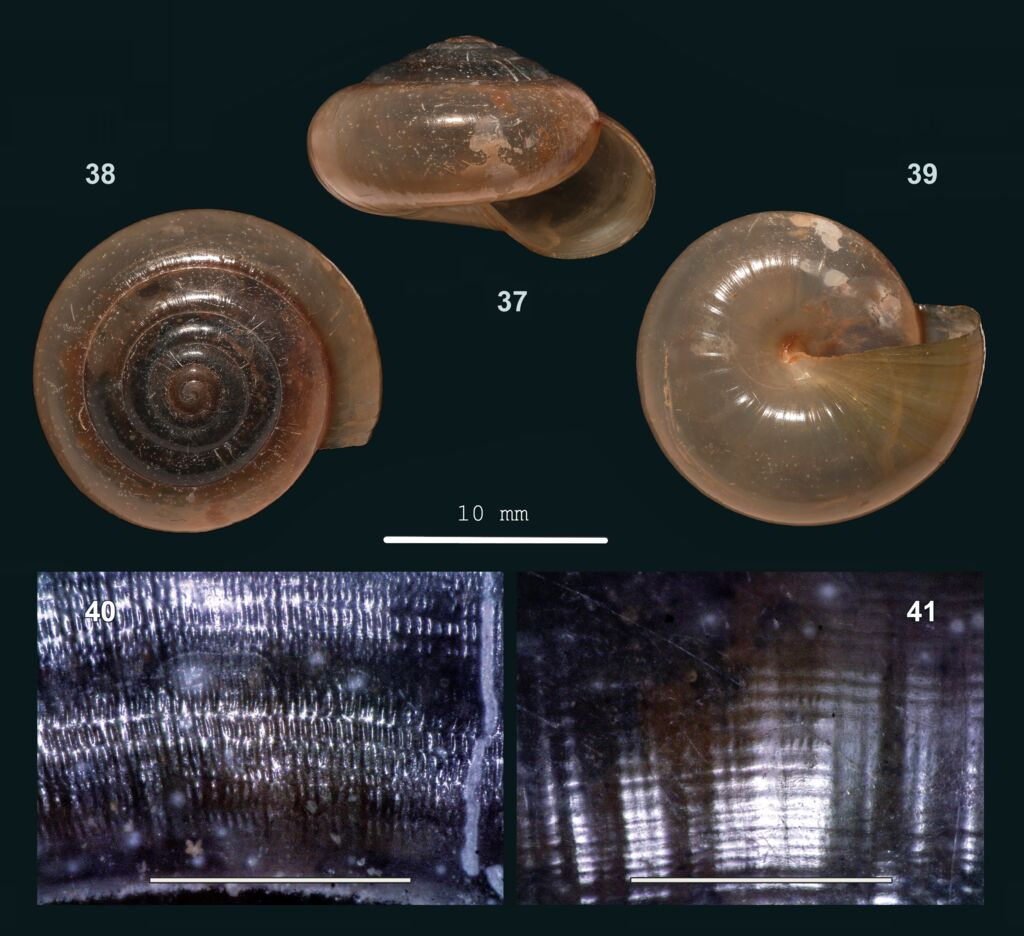
*Everettia micropleura* sp. nov.: 37 – holotype (HR L-1655/MZU.MOL 26.10), frontal view, shell height 12 mm, 38 – same shell, apical view, 39 – same shell, umbilical view, 40 – paratype (HR L-1657), upper surface of last whorl, 41 – same shell, lower surface (photographs: 37-41 - JV, 40-41 Scale: 1 mm).

Shell medium-sized to large, thin, slightly translucent, (pale) green-corneous. Surface with a silky lustre. Spire slightly to moderately raised, sides slightly convex, apex somewhat narrowly rounded. Whorls moderately convex; last whorl with periphery approx. evenly rounded. Sculpture. Protoconch approx. smooth. Teleoconch. Radial sculpture on last whorl: Inconspicuous, somewhat raised, unevenly spaced growth lines, which locally are more prominent; lines not more prominent towards suture, slightly curved very close to it or not; also with somewhat finer, dense, evenly spaced, straight, low and rounded radial riblets, most distinct above periphery, below less so, (almost) absent towards aperture. Spiral sculpture on last whorl: Above periphery continuous, rather distinct, rather widely spaced striation cutting through the crests of radial riblets, dividing these into distinctly elongated sections; below periphery, similar striation, but more prominent and cutting into shell surface where radial riblets are inconspicuous or absent. Aperture evenly rounded. Peristome neither widened, nor thickened. Umbilicus rimate or open, half-covered by peristome. Holotype (Height 12 mm; Width 18.4 mm). As shown in Fig. 12 (37-39). Paratype. As shown in Fig. 12 (40-41). Dimensions. Height 9.6–12.0 mm; width 17.4–21.0 mm; ratio height/width 0.55–0.61; diameter of first four whorls 1.5–2.0 mm, 2,8–3.4 mm, 4.9–5.7 mm and 7.7–9.0 mm, respectively; last whorl 32–40% of shell width; umbilicus up to 0.4 mm wide, up to 2% of shell width; number of whorls 5 1/4–6; height aperture 7.0–9.0 mm, 63–79% of shell height; width 8.0–10.2 mm.

#### Diagnosis

Amongst Sarawak *Everettia*, it shares radial sculpture with *E. cryptopleura* sp. nov., differs by densely placed, straight, rounded radial riblets (versus somewhat braided, flattened radial riblets) on the outer whorl above periphery, which are somewhat finer than the growth lines (versus much finer than growth lines) and by rather spaced spiral grooves (versus as dense as radial riblets) which divide radial riblets in elongated sections (versus locally dividing them in rows of flattened beads). It also differs by a narrower or rimate umbilicus: up to 0.4 mm diam. or up to 2% of shell width (versus 0.4–1.0 mm, 3–5%). It also resembles *Everettia paulbasintali* Liew, Schilthuizen & Vermeulen, 2009 (from Sabah) (see Liew et al. (2009)) in the radial sculpture, differs by being smaller (adult shells up to 21 mm wide versus 36 mm), by whorls which expand less rapidly (diameter 4^th^ whorl 7.7–9.0 mm versus 9.8–11.5 mm) and by more straight and evenly spaced radial sculpture.

#### Etymology

From μικρός (Ancient Greek) = ‘small’ and πλευρά = ‘rib’.

#### Distribution

Sarawak: Niah, Lambir, Miri, Sibuti. Elevation range: 0–100 m. Herbaceous vegetation on sandstone/shale bedrock. Endemic to Sarawak.

### Everettia platyacris sp. nov.

08329308-99E4-5670-9290-F78BA4901595

4FAC0DD9-02D5-4C10-9416-9E3D595BE20A

#### Materials

- **Type status:** Holotype. **Occurrence:** catalogNumber: MZU.MOL 26.11; occurrenceID: 4.051556° N, 114.815056° E; **Taxon:** scientificName: *Everettia platyacris*; kingdom: Animalia; phylum: Mollusca; class: Gastropoda; family: Dyakiidae; genus: Everettia; scientificNameAuthorship: Godwin-Austen, 1891; **Location:** country: Malaysia; stateProvince: Sarawak; locality: 4^th^ Div., Gunung Mulu N.P., along path from Clearwater Cave to Moonmilk Cave
- **Type status:** Paratype. **Occurrence:** catalogNumber: JV 10461/3; occurrenceID: 4.051556° N, 114.815056° E; **Taxon:** scientificName: *Everettia platyacris*; kingdom: Animalia; phylum: Mollusca; class: Gastropoda; family: Dyakiidae; genus: Everettia; scientificNameAuthorship: Godwin-Austen, 1891; **Location:** country: Malaysia; stateProvince: Sarawak; locality: 4^th^ Div., Gunung Mulu N.P., along path from Clearwater Cave to Moonmilk Cave
- **Type status:** Other material. **Occurrence:** catalogNumber: JV 10461/4; occurrenceID: 4.051556° N, 114.815056° E; **Taxon:** scientificName: *Everettia platyacris*; kingdom: Animalia; phylum: Mollusca; class: Gastropoda; family: Dyakiidae; genus: Everettia; scientificNameAuthorship: Godwin-Austen, 1891; **Location:** country: Malaysia; stateProvince: Sarawak; locality: Gunung Mulu N.P., along path from Clearwater Cave to Moonmilk Cave
- **Type status:** Other material. **Occurrence:** catalogNumber: JV 10506/2; occurrenceID: 4.072199° N, 114.876493° E; **Taxon:** scientificName: *Everettia platyacris*; kingdom: Animalia; phylum: Mollusca; class: Gastropoda; family: Dyakiidae; genus: Everettia; scientificNameAuthorship: Godwin-Austen, 1891; **Location:** country: Malaysia; stateProvince: Sarawak; locality: Gunung Mulu N.P., entrance to Sarawak Chamber
- **Type status:** Other material. **Occurrence:** catalogNumber: JV 5658/1; occurrenceID: 4.136262° N, 114.894340° E; **Taxon:** scientificName: *Everettia platyacris*; kingdom: Animalia; phylum: Mollusca; class: Gastropoda; family: Dyakiidae; genus: Everettia; scientificNameAuthorship: Godwin-Austen, 1891; **Location:** country: Malaysia; stateProvince: Sarawak; locality: Mulu N.P., Gunung Api, secondary top of crest
- **Type status:** Other material. **Occurrence:** catalogNumber: JV 5676/2; occurrenceID: 4.136572° N, 114.839595° E; **Taxon:** scientificName: *Everettia platyacris*; kingdom: Animalia; phylum: Mollusca; class: Gastropoda; family: Dyakiidae; genus: Everettia; scientificNameAuthorship: Godwin-Austen, 1891; **Location:** country: Malaysia; stateProvince: Sarawak; locality: Mulu N.P., Camp 5

#### Description

**Figure 13.**
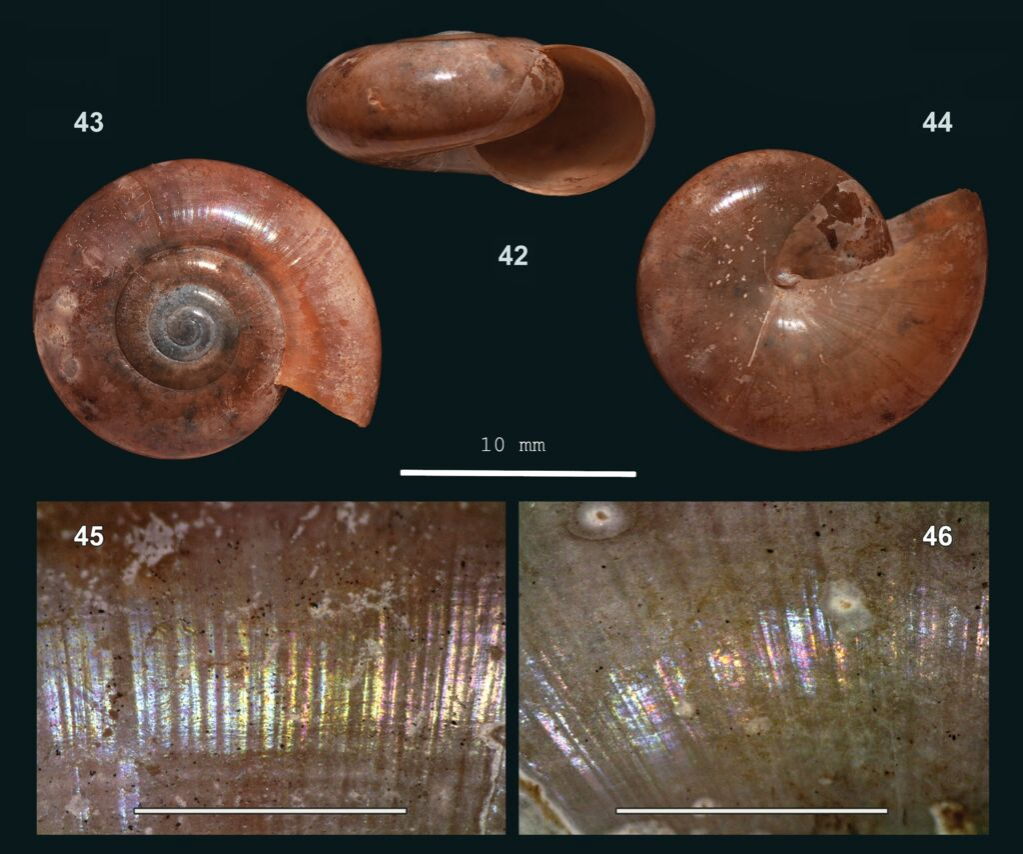
*Everettia platyacris* sp. nov.: 42 – holotype (MZU.MOL 26.11), frontal view, shell height 8 mm, 43 – same shell, apical view, 44 – same shell, umbilical view, 45 – upper surface of last whorl, 46 – same shell, lower surface (photographs; 42-46 - JV, 45-46 Scale: 1 mm).

Shell medium-sized, thin, slightly translucent, (pale brown-)corneous, umbilical region paler. Surface shiny. Spire approx. flat, apex slightly raised, narrowly rounded. Whorls: protoconch whorls slightly convex, teleoconch whorls moderately convex; last whorl more narrowly rounded at periphery. Sculpture. Protoconch approx. smooth or with some inconspicuous radial riblets. Teleoconch. Radial sculpture on last whorl: Inconspicuous, somewhat raised growth lines at uneven intervals, which locally, at usually uneven intervals, are slightly more prominent; radial sculpture hardly more distinct towards suture, not or hardly curved close to it. Spiral sculpture on last whorl: Above and below periphery with slight traces of very fine, somewhat spaced striation. Aperture evenly rounded or slightly more narrowly rounded on palatal side. Peristome neither widened, nor thickened. Umbilicus closed or rimate. Holotype (Height 8 mm; Width 16.5 mm). As shown in Fig. 13 (42-46). Dimensions. Height 5.8–8.0 mm; width 11.2–16.5 mm; ratio height/width 0.45–0.52; diameter of first four whorls 1.3–1.7 mm, 2.8–3.2 mm, 5.1–6.7 mm and 10.0–12.0 mm, respectively; last whorl 53–56% of shell width; number of whorls 3 5/8–4 1/2; height aperture 5.0–6.4 mm, 82–93% of shell height; width 6.0–8.2 mm.

#### Diagnosis

Characterised amongst Sarawak *Everettia* by the approx. flat spire, with only a slightly raised apex (versus spire slightly raised to depressed conical) and by rapidly expanding whorls (last whorl 53–56% of shell width versus 26–47%). Similar species elsewhere. Resembles *Everettia planispira* Liew, Schilthuizen & Vermeulen, 2009 (from Sabah) (see Liew et al. (2009)) in the approx. flat spire, but differs by more rapidly expanding whorls: diameter of 4^th^ whorl 10.0–12.0 mm (versus 6.0–8.6 mm), last whorl 53–56% of shell width (versus 41–48%).

#### Etymology

From πλάτη (Ancient Greek) = ‘flat’ and ἄκρις = ‘top’.

#### Distribution

Sarawak: Mulu. Elevation range: 0–1200 m. (Disturbed) primary forest on limestone bedrock. Endemic to Sarawak.

### Hypselostoma stenopyrgus sp. nov.

9F4E2CB1-8767-5762-A9E1-05E4355C446C

0374D1DA-DB4E-440A-A78D-D834BE919A5D

#### Materials

- **Type status:** Holotype. **Occurrence:** catalogNumber: MZU.MOL 18.03; occurrenceID: 0.955317° N, 110.503698° E; **Taxon:** scientificName: *Hypselostoma stenopyrgus*; kingdom: Animalia; phylum: Mollusca; class: Gastropoda; family: Hypselostomatidae; genus: Hypselostoma; scientificNameAuthorship: W. H. Benson, 1856; **Location:** country: Malaysia; stateProvince: Sarawak; locality: Serian, Gunung Silabur
- **Type status:** Paratype. **Occurrence:** catalogNumber: JV 13061/1; occurrenceID: GPS N/A; **Taxon:** scientificName: *Hypselostoma stenopyrgus*; kingdom: Animalia; phylum: Mollusca; class: Gastropoda; family: Hypselostomatidae; genus: Hypselostoma; scientificNameAuthorship: W. H. Benson, 1856; **Location:** country: Malaysia; stateProvince: Sarawak; locality: Penrissen valley, limestone range 12 km NNE of Padawan
- **Type status:** Other material. **Occurrence:** catalogNumber: JV 10213/2; occurrenceID: 3.817723° N, 113.781411° E; **Taxon:** scientificName: *Hypselostoma stenopyrgus*; kingdom: Animalia; phylum: Mollusca; class: Gastropoda; family: Hypselostomatidae; genus: Hypselostoma; scientificNameAuthorship: W. H. Benson, 1856; **Location:** country: Malaysia; stateProvince: Sarawak; locality: Niah N.P., N side of limestone area, slopes and cliffs along path to Great Cave

#### Description

**Figure 14.**
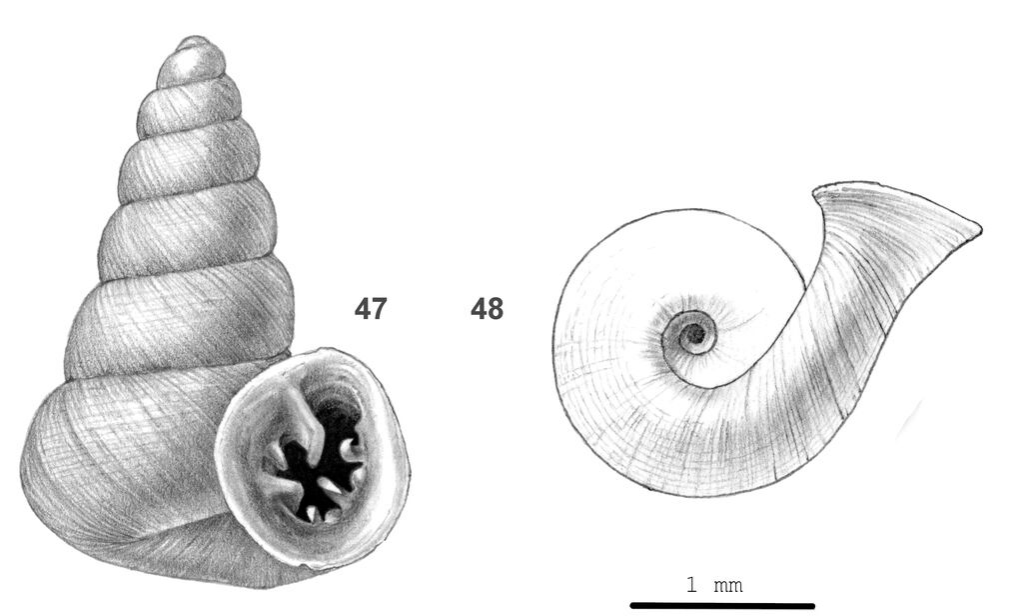
*Hypselostoma stenopyrgus* sp. nov.: 47 – paratype (JV 13061), frontal view, shell height 3.0 mm, 48 – same shell, umbilical view.

Shell very small, thin, opaque, brown. Surface dull. Spire high-conical, sides slightly concave, apex rounded. Whorls: top whorls convex, next whorls moderately convex, last rounded, a little narrowly so at periphery, edge of umbilical impression narrowly rounded; spire gradually expanded towards aperture. Suture impressed. Sculpture. Protoconch microscopically rugose. Teleoconch with unevenly spaced growth lines locally grading into low, rounded riblets. Spiral striation: widely spaced, fine, thin, low threads, subordinate to the most distinct radial sculpture only. Aperture free, parietal edge distant from spire, somewhat tilted upwards relative to spire axis, circular; teeth 6–8: A shallow angularis and a deep parietalis fused to a slightly curved lamella; 2–4 palatales (two distinct, short, hooked, with or without 1–2 small, rounded knobs below each distinct), 1 rather distinct, hooked basalis, 1 distinct, rounded columellar lamella, 1 smaller, short, rounded parietalis. Peristome spreading, thin. Holotype (Height 3.2 mm; Width 2.27 mm). Not shown. Paratype (Height 3.0 mm; Width 2.1 mm). As shown in Fig. 14 (47-48). Dimensions. Spire height excluding free part of last whorl 2.6–3.9 mm; width 1.6–2.0 mm; ratio height/width 1.65–1.82; width including free portion of last whorl 2.8–3.2 mm; umbilicus 0.4–0.8 mm wide; number of whorls 6 1/2–7 1/4; height aperture 1.1–1.4 mm; width 1.1–1.2 mm.

#### Diagnosis

Belongs to *Hypselostoma bensonianum*-group (Gojšina et al. 2025: 139), which is characterised within the genus by its spiral sculpture. *Hypselostoma stenopyrgus* shares a high spire and hooked apertural teeth with *H. benetuitum* Vermeulen, Luu, Theary & Anker, 2019, *H. chaunosalpinx* (Vermeulen, Luu, Theary & Anker, 2019) and *H. sorormajor* Gojšina, Hunyadi & Páll-Gergely, 2025, all from Indochina (see Vermeulen et al. (2019); Gojšina et al. (2025)). It differs from the first and the third by the rounded periphery of the last whorl, from the second by a slightly ascending (not descending) last part of the last whorl. Amongst Sarawak *Hypselostoma*, it is identified by its spiral sculpture and its hook-shaped apertural teeth.

#### Etymology

From στεῖνος (Ancient Greek) = narrow and πύργος = tower.

#### Distribution

Sarawak: Niah, Serian. Elevation range: 400–600 m. (Disturbed) primary forest on limestone bedrock. Endemic to Sarawak.

## Supplementary Material

XML Treatment for Hemiplecta gambut

XML Treatment for Vitrinula aulacotropis

XML Treatment for Chloritis platycephala

XML Treatment for Mariaella dussumieri

XML Treatment for Gleniskos

XML Treatment for Kaliella micradelpha

XML Treatment for Kaliella montismulu

XML Treatment for Kaliella xystrota

XML Treatment for Rahula pallidula

XML Treatment for Rahula scoliostoma

XML Treatment for Dyakia nitens

XML Treatment for Everettia cryptopleura

XML Treatment for Everettia micropleura

XML Treatment for Everettia platyacris

XML Treatment for Hypselostoma stenopyrgus
